# Independent Regulation of Type VI Secretion in *Vibrio cholerae* by TfoX and TfoY

**DOI:** 10.1016/j.celrep.2016.03.092

**Published:** 2016-04-21

**Authors:** Lisa C. Metzger, Sandrine Stutzmann, Tiziana Scrignari, Charles Van der Henst, Noémie Matthey, Melanie Blokesch

**Affiliations:** 1 Laboratory of Molecular Microbiology, Global Health Institute, School of Life Sciences, Ecole Polytechnique Fédérale de Lausanne (EPFL), 1015 Lausanne, Switzerland

**Keywords:** *Vibrio cholerae*, type VI secretion system, TfoX-like regulators, interbacterial competition, motility

## Abstract

Type VI secretion systems (T6SSs) are nanomachines used for interbacterial killing and intoxication of eukaryotes. Although *Vibrio cholerae* is a model organism for structural studies on T6SSs, the underlying regulatory network is less understood. A recent study showed that the T6SS is part of the natural competence regulon in *V. cholerae* and is activated by the regulator TfoX. Here, we identify the TfoX homolog TfoY as a second activator of the T6SS. Importantly, despite inducing the same T6SS core machinery, the overall regulons differ significantly for TfoX and TfoY. We show that TfoY does not contribute to competence induction. Instead, TfoY drives the production of T6SS-dependent and T6SS-independent toxins, together with an increased motility phenotype. Hence, we conclude that *V. cholerae* uses its sole T6SS in response to diverse cues and for distinctive outcomes: either to kill for the prey’s DNA, leading to horizontal gene transfer, or as part of a defensive escape reaction.

## Highlights

• The type VI secretion system (T6SS) of *V. cholerae* is activated by TfoX and TfoY
• Both regulators aim at different phenotypic outcomes
• TfoY drives the production of T6SS-dependent and T6SS-independent toxins
• The absence of TfoY severely impairs constitutive T6SS activity in strain V52

## Graphical Abstract

**Figure:**
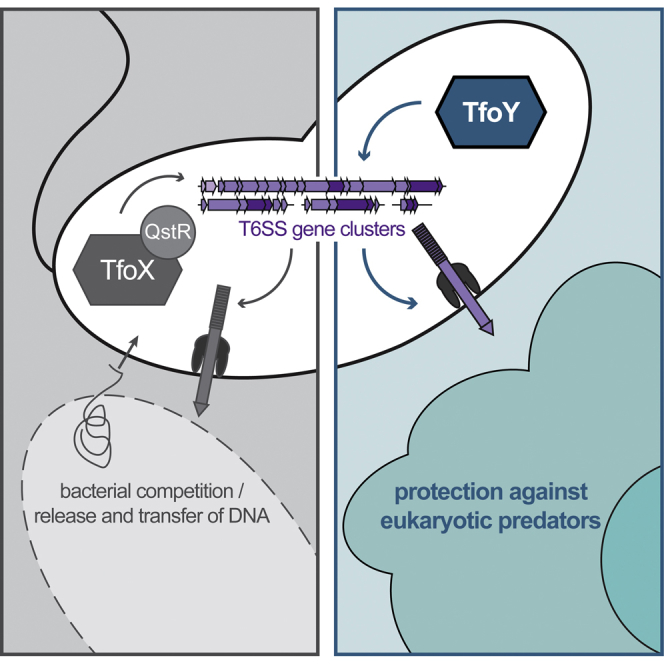

## Teaser

Metzger et al. find that the type VI secretion system (T6SS) of *Vibrio cholerae* is activated by TfoX and TfoY. Such dual regulation of the T6SS of *V. cholerae* suggests that this molecular killing device is activated by diverse environmental cues.

## Introduction

*Vibrio cholerae* is a common resident of aquatic habitats and is often found in association with chitinous surfaces (Lipp et al., 2002). Upon growth on chitinous surfaces, *V. cholerae* enters a state of natural competence for transformation (Meibom et al., 2005), which enables the bacterium to take up free DNA through its DNA-uptake machinery (Seitz and Blokesch, 2013, Seitz et al., 2014). Competence regulation in *V. cholerae* involves a complex regulatory network (Metzger and Blokesch, 2016). Briefly, upon growth to high-cell density (HCD; measured by quorum sensing [QS] and the QS regulator HapR; reviewed by Rutherford and Bassler, 2012) on chitin, *V. cholerae* produces the competence activators TfoX and QstR (Lo Scrudato and Blokesch, 2013, Meibom et al., 2005), both of which positively regulate the essential parts of the DNA-uptake machinery (Lo Scrudato and Blokesch, 2012, Lo Scrudato and Blokesch, 2013, Seitz and Blokesch, 2013). We recently demonstrated that the type VI secretion systems (T6SSs) of pandemic *V. cholerae* strains (i.e., the current seventh cholera pandemic) is part of this chitin-induced and TfoX-driven natural competence regulon and leads to the lysis of neighboring non-immune bacteria, followed by the uptake of their genetic material (Borgeaud et al., 2015). The T6SS therefore enhances horizontal gene transfer, as it frees genomic DNA from prey cells (Borgeaud et al., 2015).

T6SSs are present in ∼25% of all Gram-negative bacteria. These systems are molecular killing devices used for bacterial warfare and for the intoxication of eukaryotic cells (Ho et al., 2014, Russell et al., 2014). The T6SS consists of two main parts: a membrane-spanning part and a phage-like baseplate structure, to which a tail complex is attached (Costa et al., 2015). The latter is composed of an inner tube made of hemolysin-coregulated (Hcp) proteins, decorated on the outside with a contractile sheath structure (made of VipA and VipB proteins for *V. cholerae*). Upon contraction of the sheath, the Hcp tube and its tip proteins are propelled into neighboring cells (Basler, 2015, Ho et al., 2014). The concomitant delivery of effector toxins leads to the killing of neighboring bacteria or eukaryotic cells. Kin discrimination occurs via the production of effector-compatible immunity proteins that prevent self-destruction (Durand et al., 2014, Russell et al., 2014).

Most studies on the function and structure of the T6SS of *V. cholerae* have been performed in two non-pandemic isolates (V52 and 2740-80) that are constitutively operational with respect to T6SS activity. The rationale behind utilizing these specific strains was that current pandemic *V. cholerae* strains were considered T6SS silent under laboratory conditions (Ho et al., 2014). Indeed, until we reported chitin as an environmental inducer of the system (involving the competence regulator TfoX; Borgeaud et al., 2015), the major trigger that significantly activates T6SS in pandemic strains remained largely unknown (Ho et al., 2014).

Interestingly, *V. cholerae* and other members of the genus *Vibrio* contain an additional TfoX-like protein, designated TfoY (Pollack-Berti et al., 2010) (former name TfoX^GEMM^; Weinberg et al., 2007). Pollack-Berti et al. (2010) showed that both proteins, TfoX and TfoY, contribute to efficient natural transformation in the symbiotic bacterium *Vibrio fischeri* without being functionally identical. Moreover, these authors suggested differential regulation patterns for *tfoX* and *tfoY* of *V. fischeri*, as their transcriptional activation appeared sequential upon colonization of the light organ of the symbiotic partner (the squid) (Pollack-Berti et al., 2010, Wier et al., 2010). However, regulation of *tfoY* and any TfoY-driven transformation-independent phenotypes was not addressed.

TfoX-like proteins are commonly annotated as competence/transformation regulators. Notably, in this study we demonstrate that TfoY of *V. cholerae* does not contribute to natural competence for transformation. Instead, we identified TfoY as a second master regulator of T6SS in *V. cholerae.* T6SS activation by TfoY occurs independently of TfoX, as well as in a chitin- and QS-independent manner. Importantly, we provide evidence that TfoY is not only responsible for T6SS regulation in the most prevalent *V. cholerae* pandemic strains but also for constitutive T6SS activity in the non-pandemic strain V52. Based on comparison between the TfoX and TfoY regulons and the different phenotypes associated with them, we conclude that these two T6SS regulators initiate distinctive cell fates.

## Results

### The Competence Activator TfoX and Its Homolog TfoY

**Figure 1:**
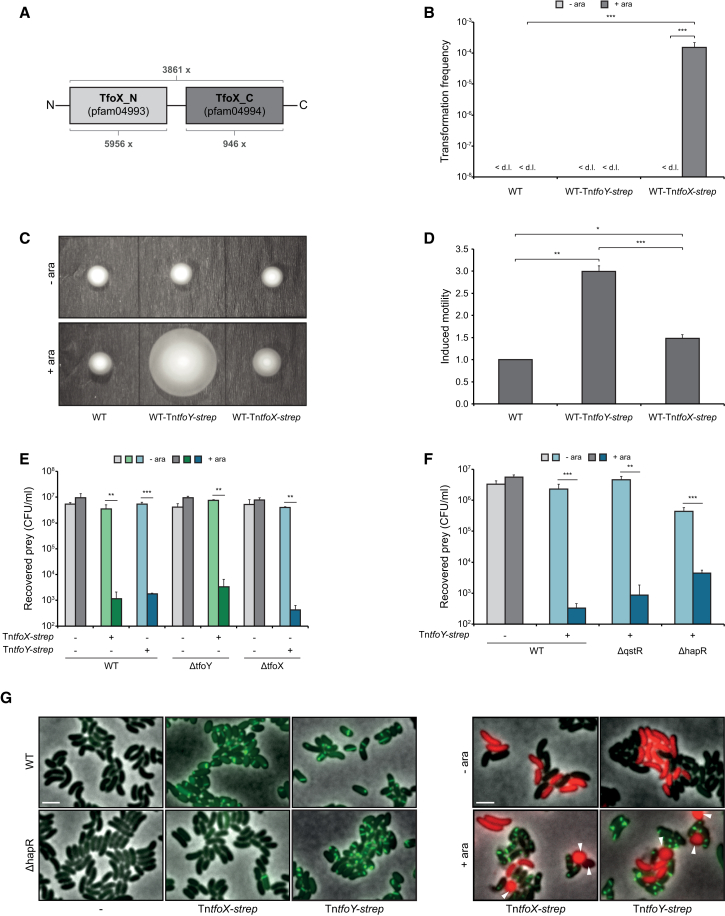
TfoX and TfoY Are Not Redundant (A) Scheme showing the two-domain structure of TfoX-like proteins. The abundance of proteins carrying either one or both domains is indicated below and above the scheme, respectively. (B–D) *V. cholerae* strains carrying a chromosomal copy of either *tfoX* or *tfoY* under the control of P_BAD_ (Tn*tfoX-strep* and Tn*tfoY-strep*) were analyzed for chitin-independent natural transformability (B) and motility on semi-solid LB agar (C and D). (B) Natural transformation is fully dependent on TfoX. The indicated strains were cultured under non-inducing (−ara) and inducing (+ara) conditions. Transformation frequencies are shown on the y axis and represent the average of three independent experiments (error bars indicate SD). <d.l., below detection limit. (C and D) TfoY induction vastly enhances surface motility. Motility was scored on soft agar without (−ara) and with (+ara) induction. Representative images are shown (C). (D) Quantification of the motility phenotype shown in (C). The average ratio between induced versus uninduced conditions is shown on the y axis based on three independent experiments (±SD). (E–G) TfoY induces T6SS-mediated interbacterial killing in a TfoX- and QS-independent manner. (E and F) Interspecies killing assay between *V. cholerae* strains and *E. coli* as prey. Indicated *V. cholerae* were co-cultured with the prey on plain LB agar (−ara) or LB agar plates supplemented with arabinose (+ara) to induce *tfoX* (green) or *tfoY* (blue). The survival of the prey is depicted as colony-forming units (CFU) per ml. Data represent the average of at least three independent biological replicates (±SD). (G) Visualization of T6SS structures (left) and T6SS-induced cell rounding of prey (right) by fluorescence microscopy. Attacked rounded prey cells are indicated by arrowheads. The brightness of the stronger TfoX-induced VipA-sfGFP signal was reduced for better visualization. Statistical significance is indicated for all panels (^∗^p < 0.05; ^∗∗^p < 0.01; ^∗∗∗^p < 0.001). See also Figure S1.

TfoX is the main activator (together with HapR and QstR) of the natural competence regulon of *V. cholerae*, which includes the T6SS (Borgeaud et al., 2015). TfoX (also referred to as Sxy in other bacteria, such as *Haemophilus influenzae*) (Redfield, 1991) and its homologs consist of two domains (TfoX-N and TfoX-C) according to the protein families database Pfam (Finn et al., 2014) (Figure 1A). Closer inspection of this organization indicated the presence of homologous domains in a plethora of other bacteria (Pfam database [v.28.0]; Finn et al., 2014) (Figure S1), suggesting a pivotal role for these protein domains.

While TfoX-driven phenotypes are well established in *V. cholerae* (Metzger and Blokesch, 2016), TfoY has not previously been studied. We therefore tested whether a *tfoY* mutant of this organism was impaired for chitin-induced natural transformation and found this was not the case (Figure S1C). Likewise, the artificial expression of *tfoY* did not result in natural transformation, in contrast to its TfoX counterpart (Figure 1B).

Although the TfoY-associated phenotypes remain unknown, a c-di-GMP riboswitch within the 5′ UTR of *tfoY* has been identified (Sudarsan et al., 2008). As c-di-GMP is an important second messenger and often involved in the transition from a planktonic (low c-di-GMP) to a sessile (e.g., biofilm at high c-di-GMP) lifestyle (Römling et al., 2013), we tested whether TfoY affected the motility of *V. cholerae*. Indeed, a *tfoY* mutant showed slightly but significantly decreased motility on soft-agar plates (Figure S1D), whereas TfoY overproduction led to a substantial increase in motility (Figure 1). This strong motility phenotype was not reproduced upon TfoX expression (Figure 1), highlighting that the two proteins are truly distinct in function in *V. cholerae*.

### TfoY Is a TfoX-Independent Regulator of T6SS

In an attempt to decipher the TfoY regulon and to potentially identify the genes involved in TfoY-mediated motility, we used an RNA-seq approach. Unexpectedly, TfoY induction also induced the T6SS gene clusters of *V. cholerae*, although not to the same extent as TfoX (Borgeaud et al., 2015) (Table S1). We therefore tested whether this TfoY-mediated increase in T6SS gene expression coincided with interbacterial killing and found this was indeed the case (Figure 1E). The killing occurred at high levels comparable to those exerted by the constitutively T6SS-active strain V52 (see below) and at levels equal to the results obtained by TfoX induction (Figure 1E). This TfoY-mediated killing was fully dependent on the presence of the T6SS (Figure S1E). Importantly, neither TfoX nor TfoY required the other to induce T6SS gene expression or to kill neighboring bacteria (Figure 1E).

To test whether TfoY-mediated T6SS induction also required co-regulation by the QS regulator HapR and QstR (Borgeaud et al., 2015), we analyzed different mutants for TfoY-induced interbacterial killing. Interestingly, and again in contrast to TfoX-dependent regulation, the TfoY-expressing mutants that lacked HapR and QstR still proficiently killed their prey (Figure 1F), indicating that QS was not essential for this regulatory circuit.

Next, we visualized the T6SS sheath protein VipA fused to sfGFP (Basler et al., 2012, Borgeaud et al., 2015) upon TfoX or TfoY induction using fluorescence microscopy. As shown in Figure 1G, both regulators were able to drive VipA production and T6SS assembly. This imaging technique also confirmed that while TfoX-mediated T6SS induction required HapR, TfoY-driven T6SS production worked independently of this QS regulator (Figure 1G). Prey rounding and lysis was likewise caused by the induction of TfoX or TfoY (Figure 1G). Altogether, we conclude that both proteins, TfoX and TfoY, induce T6SS through the involvement of distinctive co-regulatory pathways.

### VasH Differentially Contributes to TfoX- or TfoY-Mediated T6SS Induction

**Figure 2:**
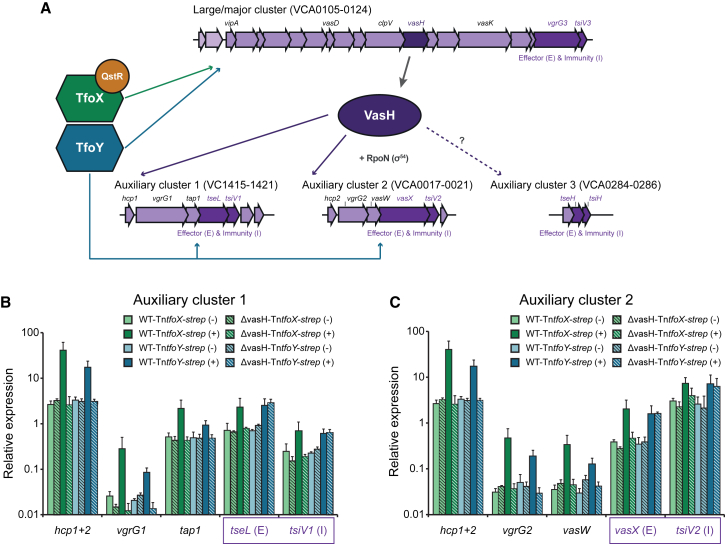
The Regulator VasH Differentially Contributes to TfoX- and TfoY-Mediated T6SS Induction (A) Scheme describing the TfoX-, TfoY-, and VasH-dependent regulation of the large and auxiliary T6SS gene clusters. (B and C) The relative expression of genes of the auxiliary T6SS gene clusters 1 (B) and 2 (C) was determined by qRT-PCR. Bacteria were grown in the absence (−) or presence (+) of arabinose to induce *tfoX* (green)/*tfoY* (blue). Data are means of at least three independent biological replicates (±SD). Effector (E)/immunity (I) genes are highlighted by boxes. See also Figure S2.

*V. cholerae* possesses a single T6SS (Pukatzki et al., 2006), encoded by a large/major gene cluster and two auxiliary clusters. A third auxiliary cluster, which encodes another set of effector/immunity proteins, was recently identified (Altindis et al., 2015) (Figure 2A).

The proteins encoded by the large/major T6SS gene cluster include a putative activator of RpoN (σ^54^) named VasH (Pukatzki et al., 2006) (Figure 2A). A *vasH* mutant of the *V. cholerae* strain V52 showed a lack of Hcp in the culture supernatant (Kitaoka et al., 2011, Pukatzki et al., 2006), and this highlights the importance of VasH in the constitutively T6SS-active strain (Dong and Mekalanos, 2012, Zheng et al., 2011).

Here, we asked whether and how VasH contributed to TfoX- and TfoY-induced T6SS expression in pandemic *V. cholerae* strains. Indeed, the protein was essential for both pathways: a *vasH* deletion strain was fully impaired for interbacterial killing upon TfoX or TfoY production (Figures S2A and S2B), even though co-regulated phenotypes, such as competence and motility, were not affected (Figures S2C and S2D). Next, we compared the expression pattern of representative genes of the four T6SS gene clusters. As shown in Figure S2E for the large T6SS cluster, VasH was dispensable for the TfoX- and TfoY-mediated expression (at least for the genes upstream of *vasH*). Moreover, with respect to auxiliary clusters 1 and 2, VasH was essential for the TfoX/TfoY-driven induction of the two *hcp* copies (Figure 2), which we also demonstrated at the protein level (Figure S2F). Likewise, VasH dependency was observed for the downstream genes of *hcp* in both auxiliary clusters upon TfoX/TfoY induction (Figure 2). However, for both clusters, the T6SS effector and immunity-encoding genes (*tseL & tsiV1* and *vasX & tsiV2*) proved to be VasH dependent only in the case of TfoX induction, while their expression was VasH independent when the system was driven by TfoY (Figure 2). Importantly, the TfoY-mediated induction of these effector-encoding genes not only occurred independently of VasH but also without a need for RpoN (Figure S2G). Finally, the recently identified effector-immunity gene pair within the third auxiliary cluster (Altindis et al., 2015) was not inducible by TfoY and was only statistically insignificantly inducible by TfoX (Figure S2H). Consistent with these expression data was the finding that TfoX- and TfoY-initiated interbacterial killing did not differ between the wild-type (WT) and a *tseH tsiH* knockout strain (Figure S2I). Hence, this T6SS effector, which most likely exhibits peptidoglycan hydrolase activity (Altindis et al., 2015), seems not to contribute to TfoX- and TfoY-induced T6SS-mediated phenotypes under the tested conditions.

### Absence of TfoY Significantly Impairs the Constitutive T6SS Activity in Strain V52

**Figure 3:**
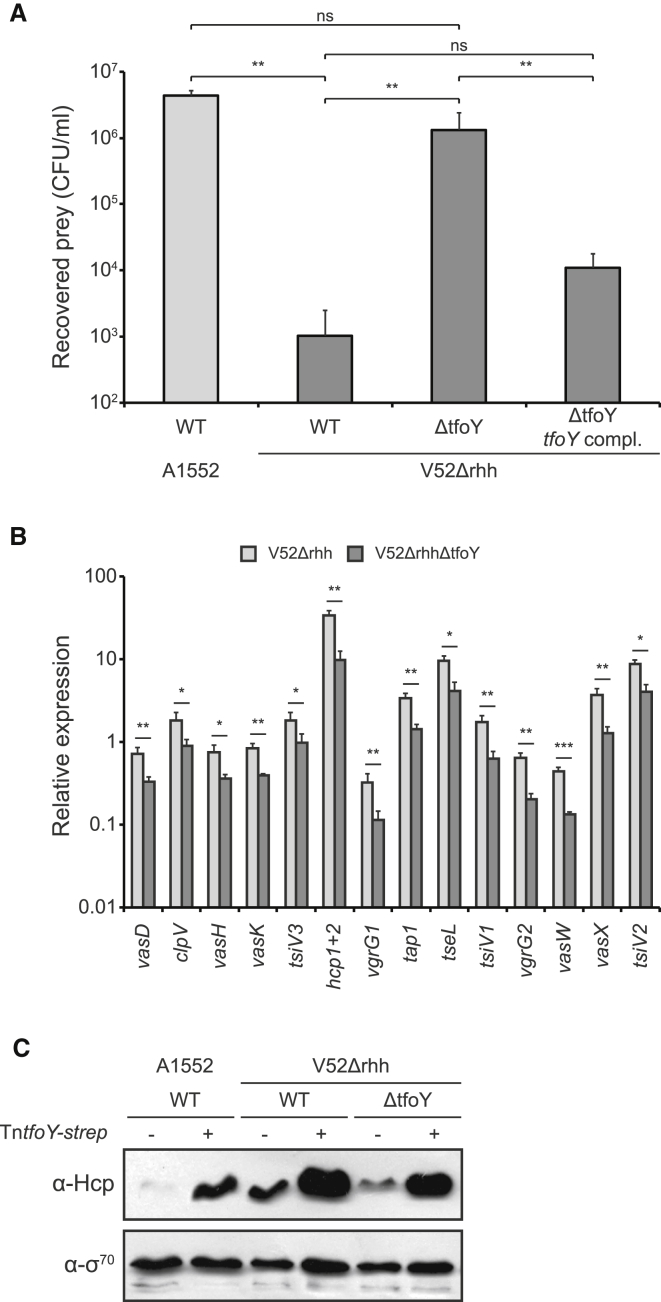
TfoY Is Primarily Responsible for Constitutive T6SS Expression in Strain V52 (A) An interbacterial killing assay with the indicated V52-derived predator strains. *E. coli* prey and the indicated predator were co-cultured on LB agar plates containing arabinose. ΔtfoY *tfoY* compl., ΔtfoY strain carrying complementing Tn*tfoY*-strep on the chromosome. Details are as in Figure 1. (B) Relative gene expression of representative T6SS genes comparing the *tfoY*-minus (V52ΔrhhΔtfoY) mutant to its parental strain (V52Δrhh). (C) Hcp production is reduced in the absence of TfoY in strain V52. Proteins of the indicated bacterial strains were detected by western blotting using Hcp-specific antibodies. The absence or presence of inducible *tfoY* (Tn*tfoY*-strep) is shown above the image. All strains were grown in the presence of the inducer (ara). Detection of σ^70^ served as the loading control. Statistical values are indicated (ns, not significant; ^∗^p < 0.05; ^∗∗^p < 0.01; ^∗∗∗^p < 0.001). See also Figure S3.

The non-pandemic strain V52 efficiently kills other Gram-negative bacteria due to its constitutive T6SS activity (MacIntyre et al., 2010) and despite a mutation that results in a premature stop codon within *hapR* (Chun et al., 2009), which renders the strain unable to enter natural competence (data not shown). We therefore wondered whether there was a link between constitutive T6SS production and the QS-independent T6SS regulator TfoY. Indeed, upon deletion of *tfoY* in strain V52, T6SS-mediated killing was almost abrogated (Figures 3A and S3A). In contrast and as expected, a deletion of *tfoX* did not interfere with T6SS-mediated killing by strain V52 (recovered prey was 7.4 × 10^3^ ± 9.2 × 10^3^ for the *tfoX* mutant compared to 4.0 × 10^3^ ± 3.9 × 10^3^ for the parental strain; average of three independent experiments ± SD). Moreover, *tfoY* deletion in strain V52 resulted in a significant decrease in T6SS gene expression as determined by qRT-PCR (Figures 3B and S3B) and accordingly in a decrease in the Hcp protein level (Figure 3C). Notably, both interbacterial killing and Hcp production could be restored in the *tfoY*-minus V52 mutant strain by re-introduction of the inducible copy of *tfoY* (Figure 3). Lastly, the lack of TfoY also significantly impaired amoebal killing (Table S2), as determined by a *Dictyostelium discoideum* plaque formation assay (Pukatzki et al., 2006). In this assay, strain V52 reduced the amoebal population to 10%, whereas the *tfoY*-minus derivative of the strain resulted in ∼95% survival (compared to a *Klebsiella*-negative control; Table S2). We therefore conclude that TfoY has a major impact on the constitutive T6SS activity in this well-studied *V. cholerae* strain V52.

### Role of c-di-GMP in TfoY Production and TfoY-Mediated Processes

We provided a first hint regarding the TfoY regulon and TfoY-associated phenotypes, but the question of how TfoY is regulated remained. As noted above, Sudarsan et al. (2008) identified a c-di-GMP riboswitch upstream *tfoY*, suggesting the involvement of the second messenger c-di-GMP in TfoY production. To elucidate the potential effect of intracellular c-di-GMP levels on TfoY activity, we engineered *V. cholerae* strains carrying an inducible copy of a c-di-GMP-producing diguanylate cyclase-encoding gene (*vdcA*) or a phosphodiesterase-encoding gene (*cdpA*) on the chromosome. The activities of the two enzymes have previously been characterized (Tamayo et al., 2008). These authors demonstrated an increase and decrease of c-di-GMP upon expression of VdcA and CdpA, respectively. When we tested these *vdcA-/cdpA-*inducible strains in a motility assay, we observed, as expected, reduced motility upon c-di-GMP increase, while degradation of the second messenger vastly enhanced motility (Figure S4A). Notably, the presence or absence of *tfoY* did not influence this outcome, indicating that TfoY is not essential for the high-motility phenotype observed under low c-di-GMP conditions.

Next, we wondered whether a change in intracellular c-di-GMP levels would interfere with bacterial killing and therefore tested the effect of the above-mentioned c-di-GMP-variable strains on co-cultured *Escherichia coli*. For this approach, we used a WT strain carrying a functional TfoY-mCherry translational fusion-encoding allele (Figure S4B) at the gene’s native locus (Table S3). As indicated in Figure S4C, a significant reduction in *E. coli* recovery was repeatedly observed upon expression of *cdpA*, which was fully dependent on the presence of *tfoY* (Figure S4D). Consistent with this finding was the detection of the TfoY-mCherry protein by western blotting upon induction of the phosphodiesterase (Figure S4E), which suggests that TfoY production is (at least in part) triggered by low c-di-GMP levels.

## Discussion

The regulatory network that drives T6SS expression in the well-studied seventh pandemic *V. cholerae* isolates has been sorely neglected in the past. Here, we addressed this lack of knowledge and demonstrated that the two regulatory proteins TfoX and TfoY significantly induce the T6SS, leading to highly efficient interbacterial killing, comparable to the killing observed in the non-pandemic constitutively T6SS-active strain V52. It was known that TfoX is produced upon growth on chitin (Meibom et al., 2005), but the production and function of TfoY remained unknown for *V. cholerae*. Based on a riboswitch associated with *tfoY* (Sudarsan et al., 2008), we tested the contribution of the secondary messenger c-di-GMP to the production of TfoY. Our data showed that decreased c-di-GMP levels enforce the production of the TfoY protein and associated phenotypes (e.g., T6SS-mediated killing). Notably, as we only observed a small but significant effect on T6SS-mediated killing (Figure S4), we hypothesize that additional signals are required for full TfoY production in nature. Indeed, a combination of transcriptional and translational control for TfoY production, similar to what has been described for TfoX (reviewed by Metzger and Blokesch, 2016), seems likely and will be addressed in future work. This impact of c-di-GMP on T6SS activity in *V. cholerae* was unexpected and opposite of that described for *Pseudomonas aeruginosa.* Indeed, in *P. aeruginosa* the H1-T6SS is produced at high c-di-GMP levels, concomitant with enhanced biofilm formation (Moscoso et al., 2011).

**Figure 4:**
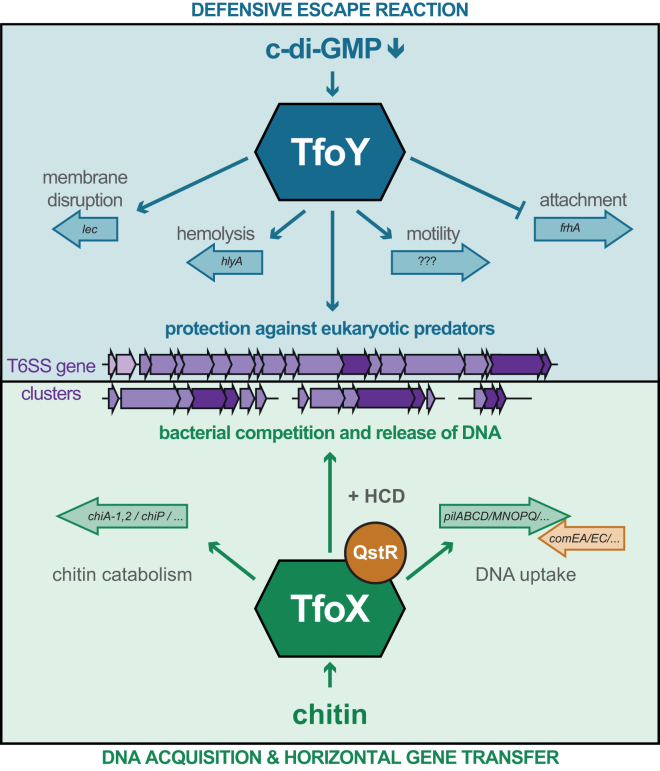
TfoY Induces a Defensive Escape Reaction Working model differentiating TfoX- and TfoY-mediated responses in *V. cholerae*. For details, see text. See also Figure S4.

With respect to the expression of the T6SS genes, Ho et al. (2014) speculated “that RpoN and VasH control only the *hcp* operons and not the main cluster suggests a two-tiered regulatory cascade. Environmental signals first need to trigger the transcription of the major cluster so that *vasH* is expressed, which subsequently activates the transcription of the *hcp* operons by RpoN.” Here, we provide evidence regarding the initial input into this two-tiered regulatory cascade. TfoX and TfoY are produced, respectively, upon reaching HCD on chitinous surfaces and through the reduction of intracellular c-di-GMP levels (Figure 4). These two regulators both initiate expression of the large T6SS cluster, including *vasH*. VasH production subsequently leads to expression of the auxiliary clusters 1 and 2 (Figure 2A). An important exception to this general regulation occurred, however: in the absence of VasH, the effector/immunity encoding genes of the auxiliary clusters 1 and 2 were still induced in a TfoY-dependent manner (Figure 2) that was not observed upon TfoX induction. We hypothesize that this superior activation by TfoY might be explained by the biological function of these two encoded effector proteins. TseL and VasX possess lipase and pore-forming activity (Dong et al., 2013, Miyata et al., 2013, Russell et al., 2013), respectively, both of which are not only functional against prokaryotes but importantly also against eukaryotes. Indeed, it has been previously shown that TseL and VasX are required to fight predation by *D. discoideum* in the T6SS-hyperactive strain V52 (Dong et al., 2013). Thus, we hypothesize that the TfoY-mediated response aims at targeting eukaryotic predators. Consistent with this idea, we demonstrated that *V. cholerae* strain V52 is severely impaired for amoebal killing in the absence of *tfoY.* In addition, a TfoY-mediated defense reaction is supported by the changed expression of several other genes, as elucidated by RNA-seq. For instance, TfoY represses the “flagellum-regulated hemagglutinin A” gene (*frhA*; 3.5-fold repression), a bacterial adhesin required for attachment (Syed et al., 2009). This detachment phenotype accompanies the enhanced motility observed upon TfoY induction (Figure 1). Moreover, TfoY led to a strong induction of hemolysin (*hlyA*; Alm et al., 1988; 8.4-fold induction) and of a lecithinase (*lec*; Fiore et al., 1997; also known as thermolabile hemolysin, *tlh*; 10-fold induction), both of which also target eukaryotic cells. Accordingly, the corresponding activities were strongly reduced in a *tfoY* mutant compared to the WT (Figure S4).

In summary, we conclude that *V. cholerae* uses two independent regulatory pathways to induce its single T6SS: first, to kill for DNA as part of its natural competence program (Borgeaud et al., 2015), and second, to kill as part of a defensive escape reaction (Figure 4). These two responses are driven by TfoX and TfoY, respectively, and provide the bacterium with the unique ability to use the same T6SS for different purposes. Further studies will show whether potential danger sensing (LeRoux et al., 2015b) contributes to full TfoY production and whether the TfoY-mediated phenotypes described in this study aim at fighting a potential threat, as previously suggested for *P. aeruginosa* (LeRoux et al., 2015a).

## Experimental Procedures

### Bacterial Strains, Plasmids, and Growth Conditions

The *V. cholerae* strains and plasmids used in this study are listed in Table S3. Detailed information on growth conditions and plasmid/strain constructions is provided in the Supplemental Experimental Procedures.

### Interbacterial Killing Assay

The interbacterial killing assay was performed as previously described (Borgeaud et al., 2015). TOP10 (Invitrogen), TOP10-TnKan (this study), and SM10λpir (Simon et al., 1983) were used as *E. coli* prey and competitors.

### RNA Sequencing and Data Analysis

Bacterial growth, RNA preparation, and DNase treatment, as well as ribodepletion, library preparation, RNA sequencing (RNA-seq), and data analysis (Microsynth) were performed as previously described (Borgeaud et al., 2015). The TfoX-induced dataset is derived from a previous study (Borgeaud et al., 2015), whereas the WT control (A1552) and the TfoY-induced dataset are part of this study (GEO: GSE79467).

### Gene Expression Analysis by qRT-PCR

qRT-PCR-based analysis of gene expression in *V. cholerae* was performed as previously described, and the relative expression values are based on normalization against *gyrA* (Lo Scrudato and Blokesch, 2012).

### SDS-PAGE and Western Blotting

Proteins from bacterial cell lysates were separated by SDS-PAGE and subjected to western blotting as previously described (Lo Scrudato and Blokesch, 2012). Specific proteins were detected using α-Hcp (Eurogentec), α-σ^70^ (BioLegend), and α-mCherry (BioVision) as the primary antibodies.

### *D. discoideum* Plaque Assay

To determine the cytotoxicity of the *V. cholerae* strain V52 and its *tfoY*-minus derivative toward the amoeba *D. discoideum* (strain DH1), a plaque assay was performed similar to the one previously described (Pukatzki et al., 2006) (see the Supplemental Experimental Procedures).

## Author Contributions

L.C.M. and M.B. designed the experiments. L.C.M., S.S., T.S., C.V.d.H., and M.B. performed the experiments. C.V.d.H. and N.M. contributed strains and ideas. L.C.M. and M.B. analyzed the data. L.C.M. and M.B. wrote the paper.

## Accession Numbers

The accession number for the WT control (A1552) and the TfoY-induced dataset reported in this paper is GEO: GSE79467.

## Supplemental Information

Document S1. Supplemental Experimental Procedures, Figures S1–S4, and Tables S1–S3

Document S2. Article plus Supplemental Information
