# Supplementary material for: Independent Regulation of Type VI Secretion in Vibrio cholerae by TfoX and TfoY
Source: Cell Rep. 2016 Apr 21;15(5):951–8. doi: 10.1016/j.celrep.2016.03.092 (PMC4858559; doi:10.1016/j.celrep.2016.03.092)
Supplement: Document S1. Supplemental Experimental Procedures, Figures S1–S4, and Tables S1–S3 [file mmc1.pdf]

**Cell Reports, Volume *15***

## Supplemental Information

# Independent Regulation of Type VI Secretion in *Vibrio cholerae* by TfoX and TfoY

**Lisa C. Metzger, Sandrine Stutzmann, Tiziana Scrignari, Charles Van der Henst, Noémie Matthey, and Melanie Blokesch**

SUPPLEMENTAL FIGURES AND LEGENDS

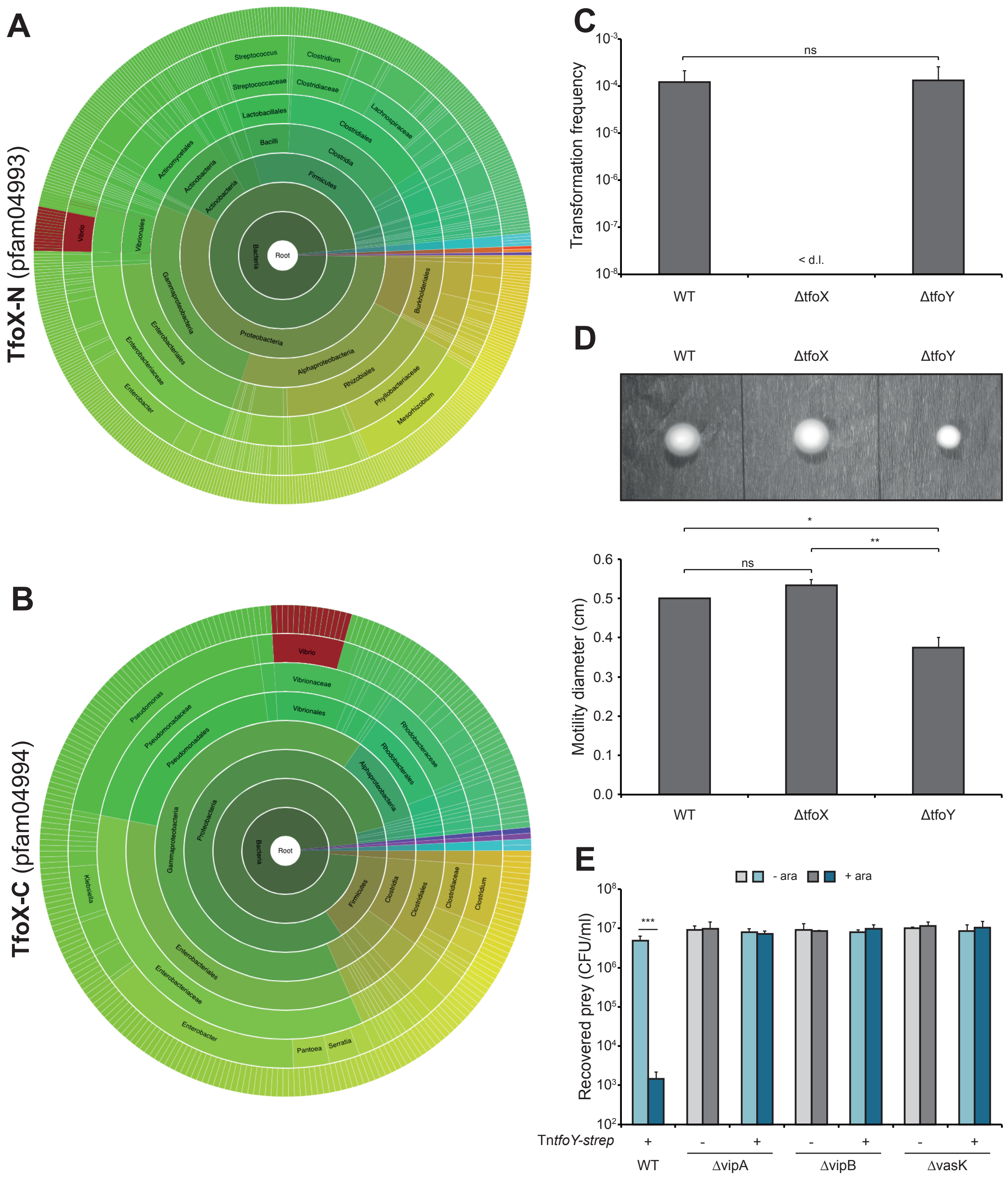


**Figure S1: Kingdom-wide distribution of TfoX-like proteins, related to Figure 1.** (A and B) Sunburst diagram showing the distribution of the two TfoX-like domains among different species. N-terminal domain of TfoX refers to PF04993 and the C-terminal domain to PF04994. Diagram derived from pfam.xfam.org (Pfam version 28.0). The segments are weighted by number of species. *Vibrio* species are highlighted in red. (C) TfoY is dispensable for chitin-induced natural transformation. Comparison of WT, ΔtfoX, and ΔtfoY strains with respect to natural transformability on chitin. Data are average transformation frequencies of at least three biological replicates (±SD). (D) TfoY influences bacterial motility. Comparison of WT, ΔtfoX, and ΔtfoY strain with respect to motility on soft agar. (E) Quantification of *E. coli* prey cells after co-incubation with the indicated *V. cholerae* predator cells (without (-ara) or with (+ara) arabinose). Details as in Figure 1.

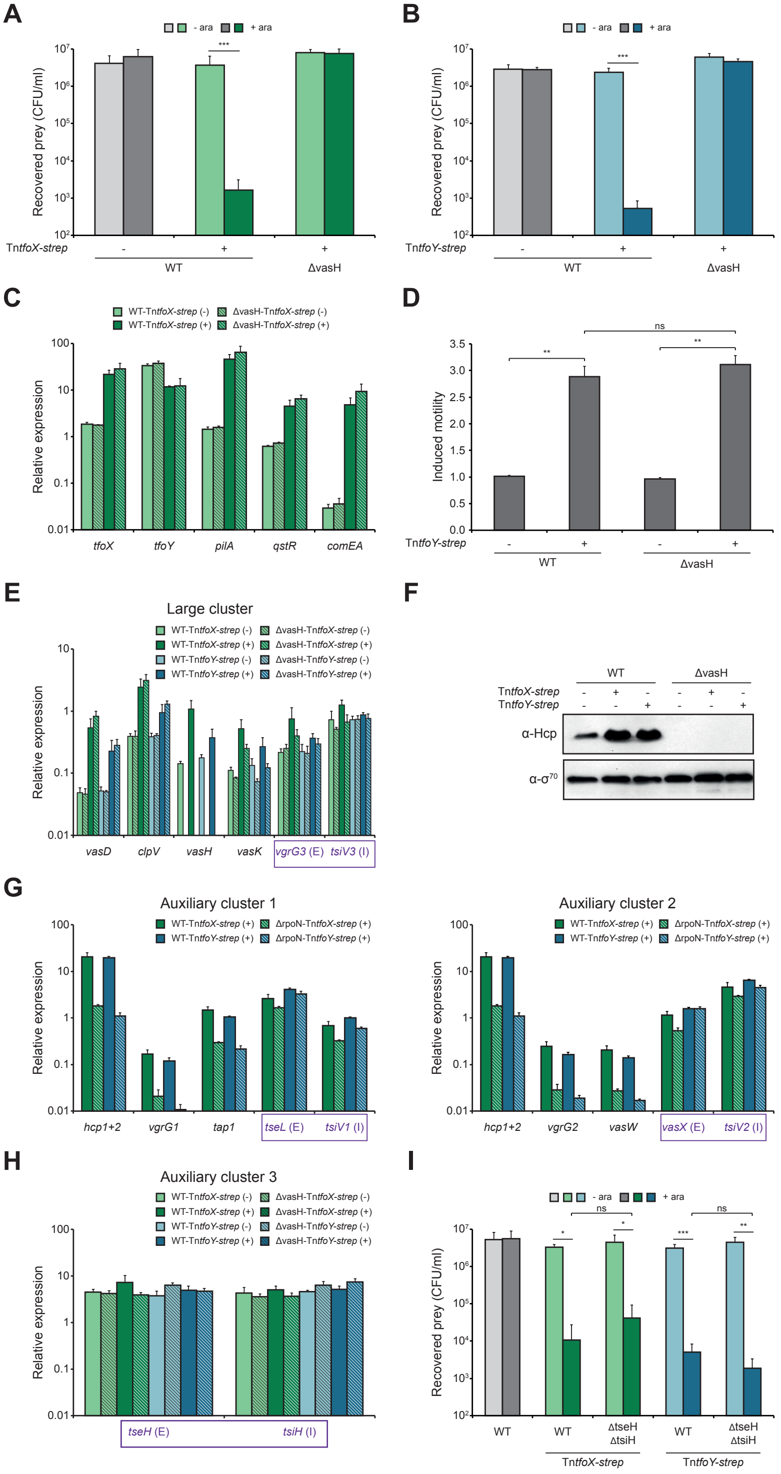


**Figure S2: The contribution of VasH to TfoX- and TfoY-dependent T6SS activity, related to Figure 2.** (A, B, and I) Interspecies killing assay between indicated *V. cholerae* as predator strains and *E. coli* prey. Quantification of surviving prey cells after the co-culturing with predator as described (see Figure 1). (C) TfoX-mediated competence induction does not rely on VasH. Transcript analysis of representative competence genes (*pilA, qstR, comEA*) in *V. cholerae* WT and a *vasH*-minus strain under *tfoX*-non-inducing (-) and *tfoX*-inducing (+) conditions. (D) TfoY-mediated motility enhancement occurs independently of VasH. Motility was scored for the indicated *V. cholerae* strains (as in Figure 1). (E) Transcript analysis in *V. cholerae* WT & ΔvasH. Relative gene expression analyzed by qRT-PCR as in Figure 2 but for the large T6SS cluster. (F) Detection of Hcp protein by western blotting. Cell lysates from the indicated *V. cholerae* strains grown to HCD (+ara) were analyzed for Hcp abundance. (G) Relative gene expression for the auxiliary clusters 1 and 2 comparing WT and an *rpoN*-minus strain. (H) Transcript analysis of the effector (E) and immunity (I) genes of auxiliary cluster 3. All experiments were performed at least three independent times, and error bars indicate standard deviations.

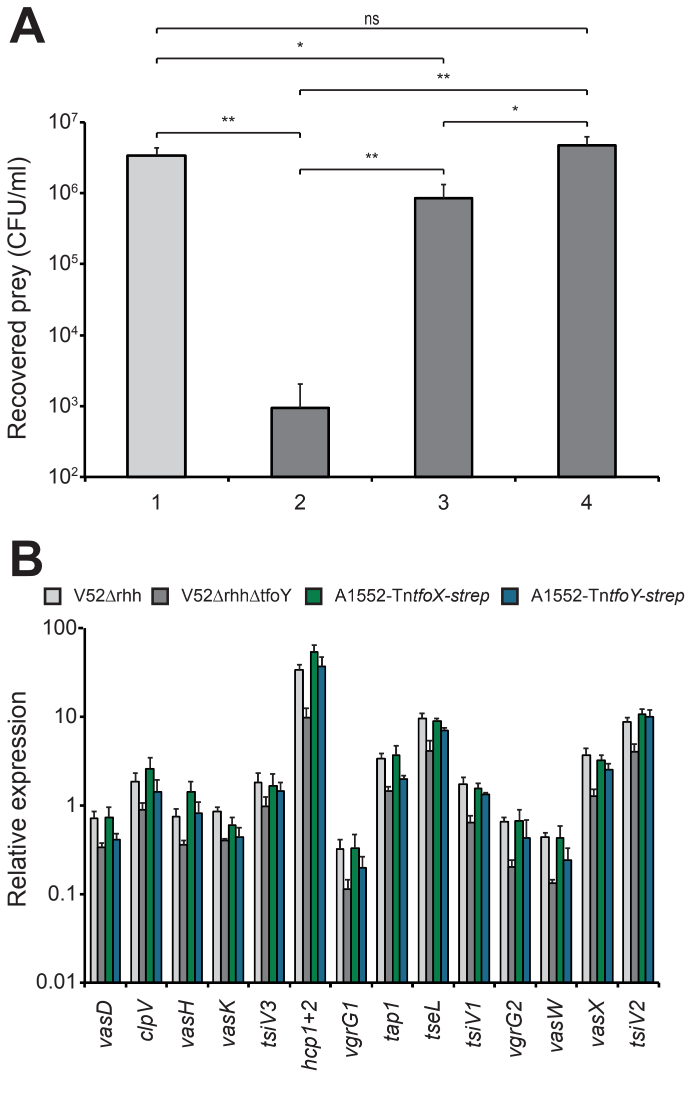


**Figure S3: The constitutive T6SS activity of strain V52 depends primarily on TfoY, related to Figure 3.** (A) A *tfoY*-minus mutant of V52 is severely impaired for interbacterial killing. Predator and prey were co-incubated on plain LB agar plates. *V. cholerae* predator strains tested (from 1 to 4 on X-axis): A1552, V52Δrhh, V52ΔrhhΔtfoY, and V52ΔvipB (T6SS-minus). Quantification of prey survival as described in Figure 1. (B) Relative expression of selected target genes as quantified by qRT-PCR in *V. cholerae* strains V52Δrhh, V52ΔrhhΔtfoY, A1552-Tn*tfoX*-strep and A1552-Tn*tfoY*-strep. Cells were grown to HCD in the presence of arabinose. Data are averages of three independent experiments (±SD).

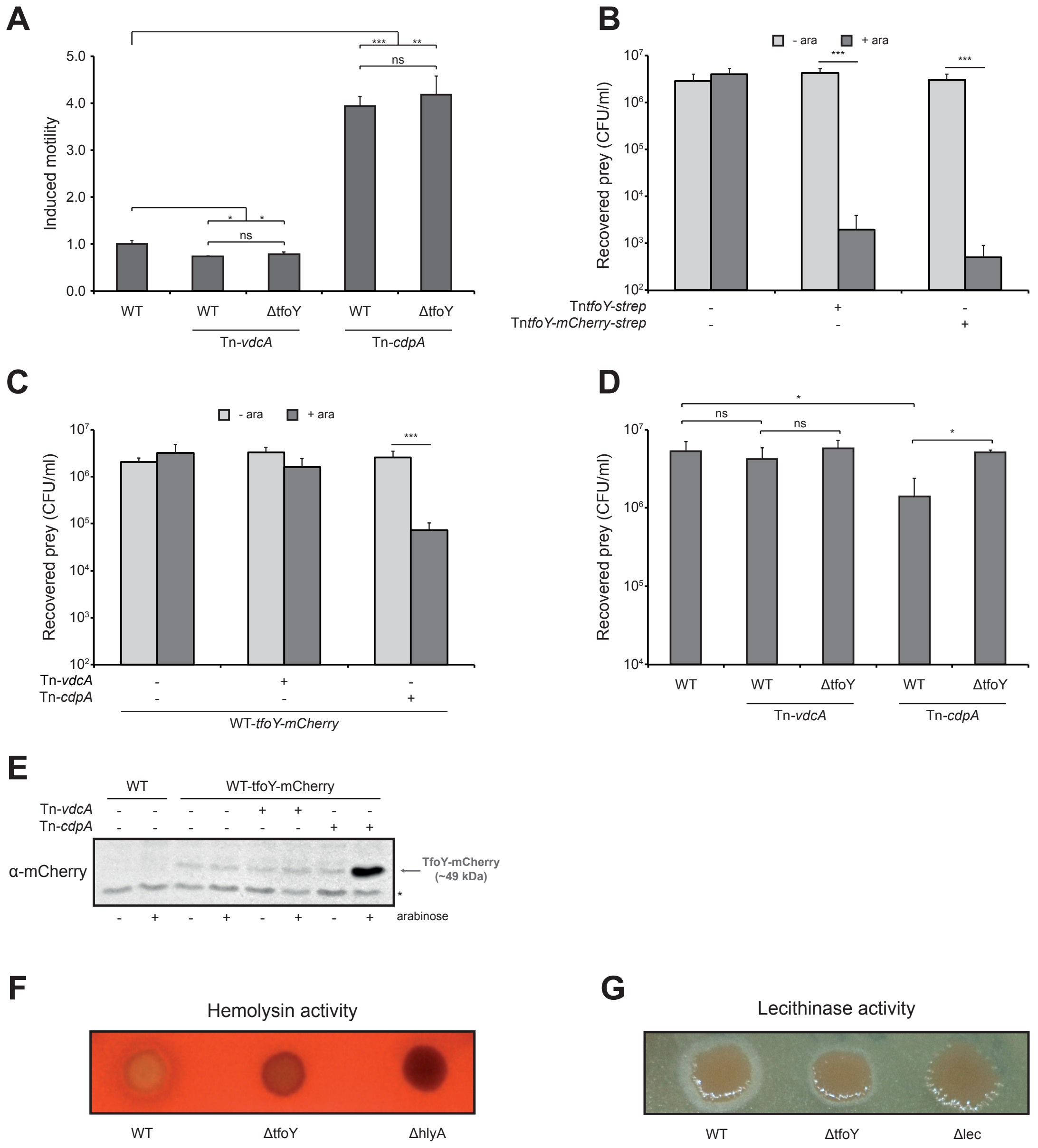


**Figure S4: TfoY-mediated phenotypes and the contribution of low c-di-GMP levels to interbacterial killing, related to Figure 4.** (A) Lowering the c-di-GMP levels within the cells increases bacterial motility. The indicated *V. cholerae* strains were spotted onto motility agar supplemented with arabinose to induce *vdcA* (a diguanylate cyclase) or *cdpA* (a phosphodiesterase) where indicated (Tn-*vdcA* and Tn-*cpdA*). Motility induction is indicated on the Y-axis. (B) The translational fusion between TfoY and mCherry remains full functionality. Interspecies killing assay between a WT, TfoY- (Tn*tfoY-strep*), and TfoY-mCherry-expressing (Tn*tfoY-mCherry-strep*) *V. cholerae* strain as predator and *E. coli* prey. (C) Low c-di-GMP levels lead to interbacterial killing. *E. coli* and the indicated *V. cholerae* strains (all carrying a gene encoding a functional translation fusion between *tfoY* and mCherry at the indigenous *tfoY* locus) were co-incubated on arabinose-lacking (-ara) or arabinose-containing (+ara) LB agar plates. (D) Expression of the phosphodiesterase *cdpA* results in TfoY-dependent interbacterial killing. *E. coli* and the indicated *V. cholerae* strains (WT or *tfoY*-minus mutant ΔtfoY) expressing a diguanylate cyclase (Tn-*vdcA*) or a phosphodiesterase (Tn-*cdpA*) were co-incubated on arabinose-containing (+ara) LB agar plates. Details as in Figure 1. (E) Detection of TfoY-mCherry produced by c-di-GMP phosphodiesterase-expressing cells. *VdcA* or *cdpA* expression was induced by arabinose where indicated. The WT strain, which lacks the *tfoY-mCherry* fusion construct, served as negative control. Western blot analysis was performed as described for Figure 3C except that mCherry-specific antibodies were utilized. A nonspecific cross-reaction (marked with an asterisk) serves as a loading control. (F) Hemolytic activity of the indicated strains was tested on blood agar plates. The WT and an *hlyA*-mutant served as the positive and negative control, respectively. (G) Activity of the lecithinase (thermolabile hemolysin) was assessed on egg yolk plates. The WT and a *lec*-minus strain served as controls.

**Table S1: RNA-seq expression data for the T6SS gene clusters in WT compared to TfoX#- and TfoY-induced cells.**

| cluster names | gene names | ID (locus tag; Heidelberg *et al*., 2000) | A1552 (WT) +ara_Exp1 | A1552 (WT) +ara_Exp2 | A1552 (WT) +ara_Exp3 | AVG A1552 (WT) +ara | A1552-TntfoX+ara_Exp1 (Borgeaud *et al*., 2015) | A1552-TntfoX+ara_Exp2 (Borgeaud *et al*., 2015) | A1552-TntfoX+ara_Exp3 (Borgeaud *et al*., 2015) | AVG A1552-TntfoX+ara (Borgeaud *et al*., 2015) | TfoX-induced (TfoX+ / WT) | A1552-TntfoY +ara_Exp1 | A1552-TntfoY +ara_Exp2 | A1552-TntfoY +ara_Exp3 | AVG A1552-TntfoY +ara | TfoY-induced (TfoY+ / WT) |
|---|---|---|---|---|---|---|---|---|---|---|---|---|---|---|---|---|
| | ***tfoX*** | **VC1153** | 8128.63 | 9285.04 | 10233.82 | 9215.83 | 29170.99 | 25425.36 | 25921.82 | 26839.39 | 2.91 | 5798.01 | 6251.69 | 6184.87 | 6078.19 | 0.66 |
| | ***tfoY*** | **VC1722** | 7491.01 | 6985.98 | 7931.44 | 7469.48 | 996.62 | 1176.54 | 1354.46 | 1175.87 | 0.16 | 31298.07 | 33495.54 | 35071.18 | 33288.26 | 4.46 |
| Auxiliary cluster 1 | *hcp1* | VC1415 | 120.58 | 137.72 | 86.41 | 114.90 | 4772.38 | 6025.73 | 3508.95 | 4769.02 | 41.51 | 3356.54 | 3977.41 | 3539.18 | 3624.38 | 31.54 |
| | *vgrG1* | VC1416 | 108.31 | 106.99 | 103.51 | 106.27 | 1263.24 | 1134.86 | 1042.16 | 1146.75 | 10.79 | 620.53 | 761.19 | 710.67 | 697.46 | 6.56 |
| | *tap1* | VC1417 | 124.66 | 97.88 | 118.81 | 113.78 | 585.63 | 471.75 | 444.47 | 500.62 | 4.40 | 470.19 | 491.62 | 527.85 | 496.56 | 4.36 |
| | *tseL* | VC1418 | 488.43 | 503.06 | 586.85 | 526.11 | 2287.81 | 2180.67 | 1810.62 | 2093.03 | 3.98 | 3503.05 | 3966.05 | 4746.79 | 4071.96 | 7.74 |
| | *tsiV1* | VC1419 | 92.99 | 124.06 | 126.01 | 114.35 | 654.32 | 921.72 | 658.51 | 744.85 | 6.51 | 794.65 | 958.46 | 1323.49 | 1025.53 | 8.97 |
| Auxiliary cluster 2 | *hcp2* | VCA0017 | 135.90 | 159.34 | 87.31 | 127.52 | 4629.17 | 5281.16 | 3434.10 | 4448.14 | 34.88 | 3432.48 | 3975.34 | 3540.46 | 3649.43 | 28.62 |
| | *vgrG2* | VCA0018 | 18.39 | 19.35 | 22.50 | 20.08 | 482.01 | 404.49 | 449.15 | 445.22 | 22.17 | 203.26 | 231.35 | 140.33 | 191.65 | 9.54 |
| | *vasW* | VCA0019 | 34.74 | 35.28 | 27.00 | 32.34 | 271.28 | 338.18 | 290.07 | 299.84 | 9.27 | 648.14 | 678.57 | 687.49 | 671.40 | 20.76 |
| | *vasX* | VCA0020 | 346.40 | 359.66 | 303.32 | 336.46 | 1744.09 | 1784.70 | 1512.36 | 1680.38 | 4.99 | 2604.85 | 2566.57 | 2630.24 | 2600.55 | 7.73 |
| | *tsiV2* | VCA0021 | 271.81 | 319.82 | 263.72 | 285.12 | 684.60 | 862.04 | 695.94 | 747.53 | 2.62 | 1007.12 | 1128.88 | 1203.76 | 1113.25 | 3.90 |
| | *PAAR1* | VCA0105 | 83.79 | 78.53 | 129.61 | 97.31 | 357.43 | 231.14 | 266.68 | 285.08 | 2.93 | 258.49 | 296.42 | 239.46 | 264.79 | 2.72 |
| | | VCA0106 | 91.96 | 100.16 | 144.01 | 112.04 | 618.23 | 434.81 | 402.36 | 485.13 | 4.33 | 316.79 | 369.75 | 418.42 | 368.32 | 3.29 |
| Large/ major cluster | *vipA* | VCA0107 | 50.07 | 59.18 | 47.70 | 52.32 | 1341.25 | 1093.18 | 1058.53 | 1164.32 | 22.25 | 418.03 | 515.38 | 525.28 | 486.23 | 9.29 |
| | *vipB* | VCA0108 | 180.86 | 187.80 | 180.91 | 183.19 | 3384.56 | 2519.80 | 2610.66 | 2838.34 | 15.49 | 1337.71 | 1502.76 | 1396.87 | 1412.45 | 7.71 |
| | | VCA0109 | 56.20 | 76.26 | 46.80 | 59.75 | 1068.81 | 747.41 | 855.02 | 890.41 | 14.90 | 493.20 | 572.18 | 431.29 | 498.89 | 8.35 |
| | *vasA* | VCA0110 | 120.58 | 118.37 | 123.31 | 120.75 | 1586.91 | 1307.26 | 1319.37 | 1404.51 | 11.63 | 730.98 | 686.83 | 757.02 | 724.94 | 6.00 |
| | *vasB* | VCA0111 | 33.72 | 35.28 | 29.70 | 32.90 | 626.38 | 591.11 | 487.74 | 568.41 | 17.28 | 257.72 | 322.24 | 294.82 | 291.60 | 8.86 |
| | *fha* | VCA0112 | 84.81 | 87.64 | 81.91 | 84.79 | 1009.43 | 842.14 | 884.26 | 911.94 | 10.76 | 520.82 | 549.46 | 608.96 | 559.75 | 6.60 |
| | *vasD* | VCA0113 | 75.62 | 66.01 | 79.21 | 73.61 | 664.80 | 632.79 | 542.72 | 613.44 | 8.33 | 418.80 | 466.84 | 442.88 | 442.84 | 6.02 |
| | *vasE* | VCA0114 | 87.88 | 96.74 | 87.31 | 90.64 | 839.44 | 1128.23 | 935.72 | 967.80 | 10.68 | 579.88 | 679.60 | 576.77 | 612.08 | 6.75 |
| | *vasF* | VCA0115 | 43.94 | 51.22 | 39.60 | 44.92 | 465.71 | 521.01 | 479.56 | 488.76 | 10.88 | 312.18 | 327.41 | 293.54 | 311.04 | 6.92 |
| | *clpV* | VCA0116 | 289.18 | 332.34 | 274.52 | 298.68 | 1832.57 | 2443.07 | 2014.14 | 2096.59 | 7.02 | 1326.97 | 1808.48 | 1462.53 | 1532.66 | 5.13 |
| | *vasH* | VCA0117 | 197.21 | 253.81 | 244.82 | 231.95 | 1305.16 | 1833.96 | 1505.34 | 1548.15 | 6.67 | 1062.34 | 1369.53 | 1180.58 | 1204.15 | 5.19 |
| | *vasI* | VCA0118 | 39.85 | 46.66 | 29.70 | 38.74 | 310.86 | 440.49 | 333.35 | 361.57 | 9.33 | 233.95 | 235.48 | 215.00 | 228.14 | 5.89 |
| | *vasJ* | VCA0119 | 139.99 | 166.17 | 117.91 | 141.36 | 773.08 | 1092.23 | 895.95 | 920.42 | 6.51 | 741.72 | 941.94 | 749.29 | 810.98 | 5.74 |
| | *vasK* | VCA0120 | 525.22 | 680.61 | 460.84 | 555.56 | 2522.99 | 3768.33 | 2609.49 | 2966.94 | 5.34 | 2324.88 | 3200.72 | 2822.07 | 2782.56 | 5.01 |
| | *vasL* | VCA0121 | 330.05 | 374.45 | 288.02 | 330.84 | 936.08 | 1589.56 | 1180.18 | 1235.27 | 3.73 | 1122.17 | 1591.58 | 1350.53 | 1354.76 | 4.09 |
| | *vasM* | VCA0122 | 38.83 | 34.14 | 33.30 | 35.43 | 64.04 | 92.83 | 101.76 | 86.21 | 2.43 | 92.81 | 124.97 | 124.88 | 114.22 | 3.22 |
| | *vgrG3* | VCA0123 | 790.89 | 931.01 | 806.46 | 842.79 | 1636.97 | 2488.54 | 1864.42 | 1996.65 | 2.37 | 1963.61 | 2550.04 | 2471.89 | 2328.51 | 2.76 |
| | *tsiV3* | VCA0124 | 345.38 | 586.15 | 477.94 | 469.82 | 826.64 | 1108.33 | 800.04 | 911.67 | 1.94 | 715.64 | 1093.76 | 1084.03 | 964.48 | 2.05 |
| Auxiliary cluster 3 | *PAAR2* | VCA0284 | 157.36 | 166.17 | 284.42 | 202.65 | 235.18 | 222.61 | 216.39 | 224.73 | 1.11 | 293.77 | 361.49 | 379.80 | 345.02 | 1.70 |
| | *tseH* | VCA0285 | 182.91 | 146.82 | 275.42 | 201.72 | 308.53 | 294.61 | 271.36 | 291.50 | 1.45 | 423.40 | 424.49 | 561.32 | 469.74 | 2.33 |
| | *tsiH* | VCA0286 | 161.45 | 129.75 | 221.42 | 170.87 | 239.84 | 306.92 | 232.76 | 259.84 | 1.52 | 505.48 | 527.77 | 690.07 | 574.44 | 3.36 |

# TfoX-induced data are from a previous study (Borgeaud et al., 2015).

**Table S2: Bacterial cytotoxicity against the amoeba *Dictyostelium discoideum***

| | Numbers of plaques# (±SD) | Statistics compared to strain V52Δrhh | Relative population size compared to *Klebsiella* negative control |
|---|---|---|---|
| V52Δrhh | 17 (± 11) | | 10.2% |
| V52ΔrhhΔtfoY | 159 (± 25) | ** ($p$ = 0.00619) | 95.2% |
| *Klebsiella sp.* | 167 (± 18) | ** ($p$ = 0.00129) | 100% |

# Average number of plaques from three independent experiments with three replicates each ± standard deviation (SD).

**Table S3:** ***V. cholerae*** **strains and plasmids used in this study**

| Strains or Plasmid | Genotype*/description | Internal strain No | Reference |
|---|---|---|---|
| A1552 (WT) | Wild-type, O1 El Tor Inaba; $Rif^R$ | MB_1 | (Yildiz and Schoolnik, 1998) |
| A1552-Tn*tfoX-strep* | A1552 containing mini-Tn7-*araC*-$P_{BAD}$-*tfoX-strep*; $Rif^R$, $Gent^R$ | MB_3420 | This study |
| A1552-Tn*tfoY* | A1552 containing mini-Tn7-*araC*-$P_{BAD}$-*tfoY*; $Rif^R$, $Gent^R$ | MB_2979 | This study |
| A1552-Tn*tfoY-strep* | A1552 containing mini-Tn7-*araC*-$P_{BAD}$-*tfoY-strep*; $Rif^R$, $Gent^R$ | MB_2978 | This study |
| A1552-Tn*tfoY-mCherry-strep* | A1552 containing mini-Tn7- *araC*-$P_{BAD}$-*tfoY-mCherry-strep*; $Rif^R$, $Gent^R$ | MB_4322 | This study |
| A1552-Tn-*vdcA* | A1552 containing mini-Tn7-*araC*-$P_{BAD}$-*vdcA*; $Rif^R$, $Gent^R$ | MB_2947 | This study |
| A1552-Tn-*cdpA* | A1552 containing mini-Tn7-*araC*-$P_{BAD}$-*cdpA*; $Rif^R$, $Gent^R$ | MB_2948 | This study |
| A1552ΔhapR | A1552ΔVC0583; $Rif^R$ | MB_3 | (Meibom et al., 2005) |
| A1552ΔhapR-Tn*tfoY-strep* | A1552ΔhapR containing mini-Tn7- *araC*-$P_{BAD}$-*tfoY-strep*; $Rif^R$, $Gent^R$ | MB_4267 | This study |
| A1552ΔhlyA | A1552 deleted for *VCA0219* (TransFLP); $Rif^R$ | MB_3935 | This study |
| A1552Δlec | A1552 deleted for *VCA0218* (TransFLP); $Rif^R$ | MB_4189 | This study |
| A1552ΔqstR | A1552ΔVC0396; $Rif^R$ | MB_600 | (Lo Scrudato and Blokesch, 2013) |
| A1552ΔqstR-Tn*tfoY-strep* | A1552ΔqstR containing mini-Tn7- *araC*-$P_{BAD}$-*tfoY-strep*; $Rif^R$, $Gent^R$ | MB_3772 | This study |
| A1552ΔrpoN | A1552 deleted for *VC2529* (TransFLP); $Rif^R$ | MB_4034 | This study |
| A1552ΔrpoN-Tn*tfoX-strep* | A1552ΔrpoN (TransFLP) containing mini-Tn7- *araC*-$P_{BAD}$-*tfoX-strep*; $Rif^R$, $Gent^R$ | MB_4088 | This study |
| A1552ΔrpoN-Tn*tfoY-strep* | A1552ΔrpoN (TransFLP) containing mini-Tn7- *araC*-$P_{BAD}$-*tfoY-strep*; $Rif^R$, $Gent^R$ | MB_4089 | This study |
| A1552ΔtfoX | A1552ΔtfoX (TransFLP); $Rif^R$ | MB_1447 | (Borgeaud et al., 2015) |
| A1552ΔtfoX-Tn*tfoY-strep* | A1552ΔtfoX (TransFLP) containing mini-Tn7- *araC*-$P_{BAD}$-*tfoY-strep*; $Rif^R$, $Gent^R$ | MB_3418 | This study |
| A1552ΔtfoY | A1552ΔVC1722 (deleted using suicide plasmid pGP704-28-SacB-Δ*tfoY*); $Rif^R$ | MB_828 | This study |
| A1552ΔtfoY-Tn*tfoX-strep* | A1552ΔtfoY containing mini-Tn7- *araC*-$P_{BAD}$-*tfoX-strep*; $Rif^R$, $Gent^R$ | MB_3419 | This study |
| A1552ΔtfoY-Tn-*vdcA* | A1552ΔtfoY containing mini-Tn7- *araC*-$P_{BAD}$-*vdcA*; $Rif^R$, $Gent^R$ | MB_3945 | This study |
| A1552ΔtfoY-Tn-*cdpA* | A1552ΔtfoY containing mini-Tn7- *araC*-$P_{BAD}$-*cdpA*; $Rif^R$, $Gent^R$ | MB_3946 | This study |
| A1552ΔtseHΔtsiH | A1552 deleted for *VCA0285* and *VCA0286* (TransFLP); $Rif^R$ | MB_4131 | This study |
| A1552ΔtseHΔtsiH-Tn*tfoX-strep* | A1552ΔtseHΔtsiH (TransFLP) containing mini-Tn7- *araC*-$P_{BAD}$-*tfoX-strep*; $Rif^R$, $Gent^R$ | MB_4185 | This study |
| A1552ΔtseHΔtsiH-Tn*tfoY-strep* | A1552ΔtseHΔtsiH (TransFLP) containing mini-Tn7- *araC*-$P_{BAD}$-*tfoY-strep*; $Rif^R$, $Gent^R$ | MB_4186 | This study |
| A1552ΔvasH | A1552 deleted for *VCA0117* (TransFLP); $Rif^R$ | MB_3928 | This study |
| A1552ΔvasH-Tn*tfoX-strep* | A1552ΔvasH (TransFLP) containing mini-Tn7- *araC*-$P_{BAD}$-*tfoX-strep* -strep; $Rif^R$, $Gent^R$ | MB_3936 | This study |
| A1552ΔvasH-Tn*tfoY-strep* | A1552ΔvasH (TransFLP) containing mini-Tn7- *araC*-$P_{BAD}$-*tfoY-strep*; $Rif^R$, $Gent^R$ | MB_3937 | This study |
| A1552ΔvasK | A1552ΔVCA0120; $Rif^R$ | MB_585 | (Borgeaud et al., 2015) |

| Strains or Plasmid | Genotype*/description | Internal strain No | Reference |
|---|---|---|---|
| A1552ΔvasK-Tn*tfoY-strep* | A1552ΔvasK containing mini-Tn7- *araC*-$P_{BAD}$-*tfoY-strep*; $Rif^R$, $Gent^R$ | MB_3578 | This study |
| A1552ΔvipA | A1552ΔVCA0107 (TransFLP); $Rif^R$ | MB_3042 | (Borgeaud et al., 2015) |
| A1552ΔvipA-Tn*tfoY-strep* | A1552ΔvipA containing mini-Tn7- *araC*-$P_{BAD}$-*tfoY-strep*; $Rif^R$, $Gent^R$ | MB_3580 | This study |
| A1552ΔvipB | A1552ΔVCA0108; $Rif^R$ | MB_598 | (Borgeaud et al., 2015) |
| A1552ΔvipB-Tn*tfoY-strep* | A1552ΔvipB containing mini-Tn7- *araC*-$P_{BAD}$-*tfoY-strep*; $Rif^R$, $Gent^R$ | MB_3579 | This study |
| A1552-tfoY-mCherry | A1552 carrying *tfoY-mCherry* translational fusion (TransFLP); $Rif^R$ | MB_4262 | This study |
| A1552-tfoY-mCherry-Tn-*vdcA* | A1552-tfoY-mCherry containing mini-Tn7- *araC*-$P_{BAD}$-*vdcA*; $Rif^R$, $Gent^R$ | MB_4324 | This study |
| A1552-tfoY-mCherry-Tn-*cdpA* | A1552-tfoY-mCherry containing mini-Tn7- *araC*-$P_{BAD}$-*cdpA*; $Rif^R$, $Gent^R$ | MB_4325 | This study |
| A1552-vipA-sfGFPv2 | A1552 carrying *vipA-sfgfp* translational fusion (TransFLP), as previously described (Borgeaud et al., 2015); this fusion version (v2) has 3 bp of the linker region removed; $Rif^R$ | MB_3909 | This study |
| A1552-vipA-sfGFPv2-Tn*tfoX-strep* | A1552-vipA-sfGFPv2 containing mini-Tn7- *araC*-$P_{BAD}$-*tfoX-strep*; $Rif^R$, $Gent^R$ | MB_3961 | This study |
| A1552-vipA-sfGFPv2-Tn*tfoY-strep* | A1552-vipA-sfGFPv2 containing mini-Tn7 *araC*-$P_{BAD}$-*tfoY-strep* $Rif^R$, $Gent^R$ | MB_3962 | This study |
| A1552ΔhapR-vipA-sfGFPv2 | A1552-vipA-sfGFPv2 (TransFLP) ΔVC0583 (deleted using suicide plasmid pGP704-28-SacB-Δ*hapR*); $Rif^R$ | MB_4217 | This study |
| A1552ΔhapR-vipA-sfGFPv2-Tn*tfoX-strep* | A1552ΔhapR-vipA-sfGFPv2 containing mini-Tn7- *araC*-$P_{BAD}$-*tfoX-strep*; $Rif^R$, $Gent^R$ | MB_4261 | This study |
| A1552ΔhapR-vipA-sfGFPv2-Tn*tfoY-strep* | A1552ΔhapR-vipA-sfGFPv2 containing mini-Tn7- *araC*-$P_{BAD}$-*tfoY-strep*; $Rif^R$, $Gent^R$ | MB_4260 | This study |
| SA5YΔvipA-*dsRED* | SA5YΔvipA-*dsRED;* $Kan^R$, $Gent^R$ | MB_3052 | (Borgeaud et al., 2015) |
| V52Δrhh | V52Δrhh; $Str^R$ | MB_3778 | (Basler et al., 2012) |
| V52Δrhh-Tn*tfoY-strep* | V52Δrhh containing mini-Tn7- *araC*-$P_{BAD}$-*tfoY-strep*; $Str^R$, $Gent^R$ | MB_4209 | This study |
| V52ΔrhhΔtfoY | V52ΔrhhΔVC1722 (deleted using suicide plasmid pGP704-28-SacB-Δ*tfoY*); ; $Str^R$ | MB_4179 | This study |
| V52ΔrhhΔtfoY-Tn*tfoY-strep* | V52ΔrhhΔtfoY containing mini-Tn7- *araC*-$P_{BAD}$-*tfoY-strep*; $Str^R$, $Gent^R$ | MB_4211 | This study |
| V52ΔrhhΔtfoX | V52ΔrhhΔVC1153 (deleted using suicide plasmid pGP704-28-SacB-Δ*tfoX*); $Str^R$ | MB_4383 | This study |
| ***E. coli* & others** | | | |
| SM10λpir | thi-1 thr leu tonA lacY supE recA::RP4-2-Tc::Mu, Kmr (λpir); $Kan^R$ | MB_647 | (Simon et al., 1983) |
| TOP10 | F- mcrA Δ(mrr-hsdRMS-mcrBC) φ80lacZΔM15 ΔlacX74 nupG recA1 araΔ139 Δ(ara-leu)7697 galE15 galK16 rpsL($Str^R$) endA1$\lambda^-$ | MB_741 | Invitrogen |
| TOP10-TnKan | TOP10 containing mini-Tn7-*aph* ($Kan^R$); $Str^R$, $Kan^R$, $Gent^R$ | MB_4119 | This study |
| DH5α | $F^-$ endA1 glnV44 thi-1 recA1 relA1 gyrA96 deoR nupG φ80lacZΔM15 Δ(lacZYA-argF) U169 hsdR17 ($r_K^-$ $m_K^+$) phoA, λ- | MB_736 | (Yanisch-Perron et al., 1985) |
| S17-1λpir | Tpr Smr recA thi pro hsdR2M1 RP4:2-Tc:Mu:Kmr Tn7 (λpir); $Str^R$ | MB_648 | (Simon et al., 1983) |

| Strains or Plasmid | Genotype*/description | Internal strain No | Reference |
|---|---|---|---|
| *Klebsiella* sp. | *Klebsiella* species; *gfp*$^{+}$ | MB_4331 | (Benghezal et al., 2006) (P. Cosson, Geneva) |
| **Plasmids** | | | |
| pBAD/myc-HisA | pBR322-derived expression vector; *araBAD* promoter ($P_{BAD}$); Amp$^{R}$ | MB_24 | Invitrogen |
| pBAD-*tfoX-strep* | *tfoX* in pBAD/Myc-HisA with C-terminal *Strep*-tagII, arabinose inducible; Amp$^{R}$ | MB_3616 | This study |
| pBAD-*tfoY* | *tfoY* in pBAD/Myc-HisA without tag, arabinose inducible; Amp$^{R}$ | MB_768 | This study |
| pBAD-*tfoY-strep* | *tfoY* in pBAD/Myc-HisA with C-terminal *Strep*-tagII, arabinose inducible; Amp$^{R}$ | MB_2945 | This study |
| pBAD-*tfoY-strep*-CSmaI | pBAD-*tfoY-strep* including SmaI site between tfoY gene and *Strep*-tagII; Amp$^{R}$ | MB_4385 | This study |
| pBAD-*tfoY-mCherry*-strep | *tfoY-mCherry* (translational fusion) in pBAD/Myc-HisA with C-terminal *Strep*-tagII, arabinose inducible; Amp$^{R}$ | MB_4386 | This study |
| pBAD-*vdcA* | *vdcA* in pBAD/Myc-HisA without tag, arabinose inducible; Amp$^{R}$ | MB_2066 | This study |
| pBAD-*cdpA* | *cdpA* in pBAD/Myc-HisA without tag, arabinose inducible; Amp$^{R}$ | MB_2065 | This study |
| pGP704-mTn7-minus SacI | pGP704 with mini-*Tn*7; Amp$^{R}$, Gent$^{R}$ | MB_645 | (Müller et al., 2007) |
| pGP704-mTn*tfoX-strep* | pGP704 with mini-Tn7 carrying *araC* and $P_{BAD}$-driven *tfoX-strep*; Amp$^{R}$, Gent$^{R}$ | MB_3664 | This study |
| pGP704-mTn*tfoY* | pGP704 with mini-Tn7 carrying *araC* and $P_{BAD}$-driven *tfoY*; Amp$^{R}$, Gent$^{R}$ | MB_2953 | This study |
| pGP704-mTn*tfoY-strep* | pGP704 with mini-Tn7 carrying *araC* and $P_{BAD}$-driven *tfoY-strep*; Amp$^{R}$, Gent$^{R}$ | MB_2941 | This study |
| pGP704-mTn*tfoY-mCherry-strep* | pGP704 with mini-Tn7 carrying *araC* and $P_{BAD}$-driven *tfoY-mCherry-strep*; Amp$^{R}$, Gent$^{R}$ | MB_4387 | This study |
| pGP704-mTn-*vdcA* | pGP704 with mini-Tn7 carrying *araC* and $P_{BAD}$-driven *vdcA*; Amp$^{R}$, Gent$^{R}$ | MB_2943 | This study |
| pGP704-mTn-*cdpA* | pGP704 with mini-Tn7 carrying *araC* and $P_{BAD}$-driven *cdpA*; Amp$^{R}$, Gent$^{R}$ | MB_2944 | This study |
| pGP704-TnKan | pGP704 with mini-Tn7 carrying *aph* (Kan$^{R}$) gene; Amp$^{R}$, Gent$^{R}$, Kan$^{R}$ | MB_4117 | This study |
| pUX-BF-13 | *ori*R6K, helper plasmid with Tn7 transposition function; Amp$^{R}$ | MB_457 | (Bao et al., 1991) |
| pGP704-Sac28 | Suicide vector, *ori*R6K, *sacB*; Amp$^{R}$ | MB_649 | (Meibom et al., 2004) |
| pGP704-28-SacB-Δ*tfoY* | pGP704-Sac28 with gene fragment resulting in a 180 bp deletion within *VC1722*; Amp$^{R}$ | MB_1133 | This study |
| p28-hapR (pGP704-28-SacB-Δ*hapR*) | pGP704-Sac28 with gene fragment resulting in a deletion from amino acid 10 downwards of *hapR;* Amp$^{R}$ | MB_1106 | (Meibom et al., 2005) |
| p28-tfoX (pGP704-28-SacB-Δ*tfoX*) | pGP704-Sac28 with a gene fragment resulting in a deletion of amino acids 6-196 of TfoX; Amp$^{R}$ | MB_1013 | This study |
| pBR-FRT-Kan-FRT2 | pBR322 derivative containing improved FRT-*aph*-FRT cassette, used as template for TransFLP; Amp$^{R}$, Kan$^{R}$ | MB_3782 | This study |
| pBR-FRT-Cat-FRT2 | pBR322 derivative containing improved FRT-*cat*-FRT cassette, used as template for TransFLP; Amp$^{R}$, Cm$^{R}$ | MB_3783 | This study |

*VC numbers according to (Heidelberg et al., 2000).

## EXTENDED EXPERIMENTAL PROCEDURES

### Bacterial strains, plasmids, and growth conditions

*V. cholerae* strains and plasmids are listed in Table S3. *Escherichia coli* strains DH5α (Yanisch-Perron et al., 1985), TOP10 (Invitrogen), and S17-1λpir (Simon et al., 1983) were used for cloning purposes and served as donors in bacterial mating experiments, respectively. A V52Δrhh (Δ*rtxA* Δ*hlyA* Δ*hapA*) strain served as the parental strain for T6SS activity and amoebal killing (see below), in accordance with previous protocols (Bachmann et al., 2015; Miyata et al., 2011).

*V. cholerae* and *E. coli* strains were grown aerobically in LB medium or on LB agar plates at 30°C or 37°C unless otherwise stated. LB motility soft agar plates contained reduced agar concentrations (0.3%) compared to standard LB agar plates (1.5%). When required, arabinose (for the expression of *tfoX*, *tfoY, tfoY-mCherry, vdcA*, and *cpdA* under the control of the $P_{BAD}$ promoter) or antibiotics were added to the growth medium at the following concentrations: L-arabinose 0.02% or 0.2%, ampicillin 50 μg/ml or 100 μg/ml, kanamycin 75 μg/ml, streptomycin 100 μg/ml, chloramphenicol 2.5 μg/ml, and gentamicin 50 μg/ml. For *E. coli* counter-selection after tri-parental mating with *V. cholerae,* Thiosulfate Citrate Bile Salts Sucrose (TCBS) agar plates were used and prepared following the manufactures' instructions (Sigma-Aldrich/Fluka, Buchs, Switzerland). Half-concentration defined artificial seawater (0.5x DASW; (Meibom et al., 2005)) was used for chitin-induced natural transformation experiments.

### Genetic engineering of strains and plasmids

All DNA manipulations were performed according to standard molecular biology protocols. Enzymes were purchased from the following companies and used as recommended by the manufacturer: Pwo polymerase (Roche), Taq polymerase (Promega), and restriction enzymes (New England Biolabs). Genetically engineered bacterial strains and plasmids were screened by colony PCR followed by Sanger sequencing (Microsynth, Switzerland).

To genetically modify *V. cholerae* strains, a gene disruption method based on the counter-selectable suicide plasmid pGP704-Sac28 (Meibom et al., 2004) or a transformation-based genetic engineering method (TransFLP; (Blokesch, 2012; Borgeaud and Blokesch, 2013; De Souza Silva and Blokesch, 2010)) was utilized. The latter technique was also used to replace the *tfoY* gene of *V. cholerae* A1552 with a translational *tfoY-mCherry* fusion allele at the gene's native locus. The plasmids pBR-FRT-Kan-FRT2 and pBR-FRT-Cat-FRT2 served as templates for the addition of the flippable antibiotics cassette.

Plasmid pBAD-*tfoX-strep* was constructed by amplification of the *tfoX* gene with *Strep-tagII*-encoding primers using the genomic DNA (gDNA) of *V. cholerae* strain A1552 as the template. The restriction enzyme-digested PCR product was cloned into the appropriately digested plasmid pBAD/MycHisA (Table S3). The *tfoY* gene was amplified using gDNA from *V. cholerae* strain A1552 and cloned after restriction enzyme digestion into the plasmid pBAD/MycHisA, resulting in plasmid pBAD-*tfoY* (Table S3). The same strategy was employed to obtain plasmids pBAD-*vdcA* and pBAD-*cdpA* (Table S3). The *Strep-tagII*-encoding sequence was added to *tfoY* by inverse PCR using overlapping oligonucleotides. The template was subsequently digested by *Dpn*I, and the non-digested PCR fragment was column-purified and used directly to transform chemically competent *E. coli* cells.

The plasmid pBAD-*tfoY-mCherry-strep* was constructed in two steps. First, a *Sma*I restriction site was introduced in-between the *tfoY* open reading frame and the *Strep-tagII* by inverse PCR on plasmid pBAD-*tfoY-strep,* as described above, resulting in plasmid pBAD-*tfoY-strep*-CSmaI. Subsequently, the mCherry-encoding gene was amplified using phosphorylated primers and cloned into the *Sma*I-digested pBAD-*tfoY-strep*-CSmaI plasmid.

The fragments containing *araC*, the arabinose-inducible promoter $P_{BAD}$, and the *tfoX-strep*, *tfoY-strep, tfoY-mCherry-strep*, *vdcA* or *cdpA* gene were amplified from the corresponding pBAD plasmids (Table S3) and cloned into the mini-Tn7-containing delivery plasmid (Table S3). For the insertion of this mini-Tn7 transposon into the *V. cholerae* chromosome, a triparental mating strategy was employed (Bao et al., 1991). The donor plasmids are indicated in Table S3.

### Natural transformation assays

The natural transformability of *V. cholerae* strains grown on chitin surfaces was determined as previously described (De Souza Silva and Blokesch, 2010; Marvig and Blokesch, 2010). To test for chitin-independent transformation, the strains carried an arabinose-inducible copy of *tfoX-strep* (e.g., including the *Strep*-tagII-encoding sequence) or *tfoY-strep* on the chromosome. The assay was performed as previously described (Lo Scrudato and Blokesch, 2012). The gDNA of A1552-lacZ-Kan (Marvig and Blokesch, 2010) served as the transforming material in all transformation assays. Transformation frequencies were calculated as the number of transformants divided by the total number of colony-forming units (CFU). At least three independent biological replicates were performed for

each experiment, and averages of all experiments are indicated in the figure (±SD). Statistical analyses of transformation frequencies were performed on log-transformed data using a two-tailed Student's *t*-test. For values below the detection limit, the detection limit value was utilized for statistical calculations.

**Interbacterial killing assay**

The interbacterial killing assay was performed following a previously established protocol (Borgeaud et al., 2015). Briefly, the predatory *V. cholerae* strain and the *E. coli* prey were mixed at a 10:1 ratio and spotted onto membrane filters on pre-warmed LB agar plates (± 0.2% ara). After incubation at 37°C for 4 h, the bacteria were resuspended from the filters, and serial dilutions were spotted onto plain LB (growth control) and antibiotic-containing LB agar plates (to select for the respective *E. coli* prey strains) to enumerate colony-forming units (CFU/ml). At least three biological experiments were performed, and averages of these independent replicates are given throughout the manuscript. Statistically significant differences were determined by the two-tailed Student's *t*-test on log-transformed data. In case the *E. coli* CFUs were below the detection limit of 200 CFU/ml, the value was set to the detection limit to allow statistical analysis.

**Motility assay**

To assess the motility of *V. cholerae,* 2 μl of the relevant overnight culture was carefully spotted onto freshly prepared LB motility agar plates and incubated at room temperature for 24 h. The next day, the swarming diameter was scored. The motility induction was calculated by dividing the swarming diameter under induced versus uninduced conditions. All experiments were repeated several times independently ($n \geq 3$), and averages of all experiments (± standard deviation) are provided. A two-tailed Student's *t*-test was performed for statistical analyses.

**Gene expression analysis by qRT-PCR**

Quantitative reverse transcription PCR (qRT-PCR)-based transcript scoring in *V. cholerae* was performed following a previously published protocol (Lo Scrudato and Blokesch, 2012). Averages of at least three biologically independent experiments (± standard deviation) are provided.

**SDS-PAGE and Western Blotting**

For the preparation of cell lysates, bacteria were cultivated in LB medium without or with arabinose, as indicated. After harvesting, the bacterial cells were resuspended in an appropriate volume of Laemmli buffer to adjust for the total number of bacteria (based on measurement of the optical density at 600 nm; $OD_{600}$) and boiled for 15 minutes. Proteins were separated by sodium dodecyl sulfate (SDS)-polyacrylamide gel electrophoresis and subjected to western blotting as previously described (Lo Scrudato and Blokesch, 2012). Primary antibodies against Hcp (see below), *E. coli* Sigma 70 (BioLegend, USA distributed via Brunschwig, Switzerland), or mCherry (BioVision, USA distributed via LubioScience, Switzerland) were diluted at 1:5'000. Goat anti-rabbit horseradish peroxidase (HRP) (diluted 1:20'000; Sigma-Aldrich, Switzerland) and goat anti-mouse HRP (diluted 1:5'000, Sigma-Aldrich, Switzerland) were used as secondary antibodies. Lumi-Light$^{PLUS}$ western blotting substrate (Roche, Switzerland) was used as the HRP substrate and the signals were detected on chemiluminescence-detecting films (Amersham Hyperfilm ECL, GE Healthcare).

**Generation of antibodies against Hcp**

Anti-Hcp antibodies were raised in rabbits against synthetic peptides (Eurogentec, Belgium). To exclude potential cross-reactions with proteins migrating towards the same position as the target protein, the antibody was tested against the *hcp1 hcp2* double knockout strain using western blotting (data not shown).

**Epifluorescence microscopy and image analysis**

Wide-field microscopy images were acquired using an epifluorescence microscope (Zeiss Axio Imager M2; details are provided elsewhere (Lo Scrudato and Blokesch, 2012)). The bacterial cultures were grown in LB medium (±ara) and then mounted onto agarose pads. Image analysis and processing were performed using the Zeiss AxioVision software, ImageJ, and Adobe Illustrator. For comparisons between different culture conditions (e.g., WT versus *hapR*-minus strains grown in the absence or presence of inducible *tfoX* or *tfoY*), the images were acquired using the exact same exposure time. However, the brightness was decreased for the TfoX-induced vipA-sfGFP signal for a better representation. For intraspecies killing, the two strains (predator and non-immune prey) were mixed at a ratio of 1:1 before being applied to an agarose pad. The mixed communities were incubated for 2 h at 24°C before imaging.

***Dictyostelium discoideum* plaque assay**

To determine the cytotoxicity of *V. cholerae* strain V52 (V52Δrhh) toward *D. discoideum*, an amoebal plaque assay was performed following a previously described protocol (Pukatzki et al., 2006) with slight modifications. Briefly, bacteria were cultured overnight in LB medium at 30°C and harvested by centrifugation. The cell pellet was resuspended in SorC buffer and diluted to an $OD_{600}$ of 5.5. *D. discoideum* cells were detached, collected by centrifugation, resuspended in SorC buffer, and added to the bacterial suspension to a concentration of 5 x $10^2$ amoebal cells per ml. Subsequently, 200 µl of the homogenate was plated on agar plates made of 5-fold diluted SM medium (SM medium according to (Sussman, 1987)). Plates were incubated for 3 to 5 days at 24°C, followed by the enumeration of the number of *D. discoideum* plaques. Three independent experiments were performed, each containing three technical replicates, and a two-tailed Student's *t*-test was performed to ascertain statistical significance.

**Hemolysin activity on blood agar plates**

The hemolytic activity of *V. cholerae* was assayed using trypticase soy agar containing 5% sheep blood (BD, Allschwil, Switzerland) supplemented with 1 mM EDTA. The respective overnight cultures were spotted onto the plates and incubated at 30°C for 24 h to 30 h. The WT and a *hlyA*-minus mutant served as the positive and negative control, respectively.

**Lecithinase / thermolabile hemolysin activity on egg yolk plates**

To assess the activity of the lecithinase (also known as thermolabile hemolysin/phospholipase), *V. cholerae* bacteria grown overnight were spotted onto egg yolk-containing agar plates and incubated at 30°C for 72 h. Plates were freshly prepared as follows: a 30% egg yolk (Sigma-Aldrich/Fluka, Buchs, Switzerland) suspension in sterile water was prepared and subsequently diluted 1:20 in autoclaved, warm LB agar before plates were poured. The WT and a *lec*-minus mutant strain served as the positive and negative control, respectively.

**SUPPLEMENTAL REFERENCES**


Bachmann, V., Kostiuk, B., Unterweger, D., Diaz-Satizabal, L., Ogg, S., and Pukatzki, S. (2015). Bile Salts Modulate the Mucin-Activated Type VI Secretion System of Pandemic *Vibrio cholerae*. PLoS Negl. Trop. Dis. *9*, e0004031.

Bao, Y., Lies, D.P., Fu, H., and Roberts, G.P. (1991). An improved Tn*7*-based system for the single-copy insertion of cloned genes into chromosomes of Gram-negative bacteria. Gene *109*, 167-168.

Basler, M., Pilhofer, M., Henderson, G.P., Jensen, G.J., and Mekalanos, J.J. (2012). Type VI secretion requires a dynamic contractile phage tail-like structure. Nature *483*, 182-186.

Benghezal, M., Fauvarque, M.O., Tournebize, R., Froquet, R., Marchetti, A., Bergeret, E., Lardy, B., Klein, G., Sansonetti, P., Charette, S.J.*, et al.* (2006). Specific host genes required for the killing of *Klebsiella* bacteria by phagocytes. Cell. Microbiol *8*, 139-148.

Blokesch, M. (2012). TransFLP – a method to genetically modify *V. cholerae* based on natural transformation and FLP-recombination. J. Vis. Exp. *68*, e3761.

Borgeaud, S., and Blokesch, M. (2013). Overexpression of the *tcp* gene cluster using the T7 RNA polymerase/promoter system and natural transformation-mediated genetic engineering of *Vibrio cholerae*. PLoS One *8*, e53952.

Borgeaud, S., Metzger, L.C., Scrignari, T., and Blokesch, M. (2015). The type VI secretion system of *Vibrio cholerae* fosters horizontal gene transfer. Science *347*, 63-67.

De Souza Silva, O., and Blokesch, M. (2010). Genetic manipulation of *Vibrio cholerae* by combining natural transformation with FLP recombination. Plasmid *64*, 186-195.

Heidelberg, J.F., Eisen, J.A., Nelson, W.C., Clayton, R.A., Gwinn, M.L., Dodson, R.J., Haft, D.H., Hickey, E.K., Peterson, J.D., Umayam, L.*, et al.* (2000). DNA sequence of both chromosomes of the cholera pathogen *Vibrio cholerae*. Nature *406*, 477-483.

Lo Scrudato, M., and Blokesch, M. (2012). The regulatory network of natural competence and transformation of *Vibrio cholerae*. PLoS Genet. *8*, e1002778.

Lo Scrudato, M., and Blokesch, M. (2013). A transcriptional regulator linking quorum sensing and chitin induction to render *Vibrio cholerae* naturally transformable. Nucleic Acids Res. *41*, 3644-3658.

Marvig, R.L., and Blokesch, M. (2010). Natural transformation of *Vibrio cholerae* as a tool-optimizing the procedure. BMC Microbiol. *10*, 155.

Meibom, K.L., Blokesch, M., Dolganov, N.A., Wu, C.-Y., and Schoolnik, G.K. (2005). Chitin induces natural competence in *Vibrio cholerae*. Science *310*, 1824-1827.

Meibom, K.L., Li, X.B., Nielsen, A.T., Wu, C.Y., Roseman, S., and Schoolnik, G.K. (2004). The *Vibrio cholerae* chitin utilization program. Proc. Natl. Acad. Sci. USA *101*, 2524-2529.

Miyata, S.T., Kitaoka, M., Brooks, T.M., McAuley, S.B., and Pukatzki, S. (2011). *Vibrio cholerae* requires the type VI secretion system virulence factor VasX to kill Dictyostelium discoideum. Infect. Immun. *79*, 2941-2949.

Müller, J., Miller, M.C., Nielsen, A.T., Schoolnik, G.K., and Spormann, A.M. (2007). *vpsA*- and *luxO*-independent biofilms of *Vibrio cholerae*. FEMS Microbiol. Lett. *275*, 199-206.

Pukatzki, S., Ma, A.T., Sturtevant, D., Krastins, B., Sarracino, D., Nelson, W.C., Heidelberg, J.F., and Mekalanos, J.J. (2006). Identification of a conserved bacterial protein secretion system in *Vibrio cholerae* using the *Dictyostelium* host model system. Proc. Natl. Acad. Sci. USA *103*, 1528-1533.

Simon, R., Priefer, U., and Pühler, A. (1983). A broad host range mobilization system for *in vivo* genetic engineering: transposon mutagenesis in Gram negative bacteria. Nat. Biotechnol. *1*, 784-791.

Sussman, M. (1987). Cultivation and synchronous morphogenesis of *Dictyostelium* under controlled experimental conditions. Methods Cell Biol. *28*, 9-29.

Yanisch-Perron, C., Vieira, J., and Messing, J. (1985). Improved M13 phage cloning vectors and host strains: nucleotide sequences of the M13mp18 and pUC19 vectors. Gene *33*, 103-119.

Yildiz, F.H., and Schoolnik, G.K. (1998). Role of *rpoS* in stress survival and virulence of *Vibrio cholerae*. J. Bacteriol. *180*, 773-784.
