# Supplementary material for: Independent Regulation of Type VI Secretion in Vibrio cholerae by TfoX and TfoY
Source: Cell Rep. 2016 Apr 21;15(5):951–8. doi: 10.1016/j.celrep.2016.03.092 (PMC4858559; doi:10.1016/j.celrep.2016.03.092)
Supplement: Document S2. Article plus Supplemental Information [file mmc2.pdf]

Cell Reports

Report

Independent Regulation of Type VI Secretion in *Vibrio cholerae* by TfoX and TfoY

Graphical Abstract

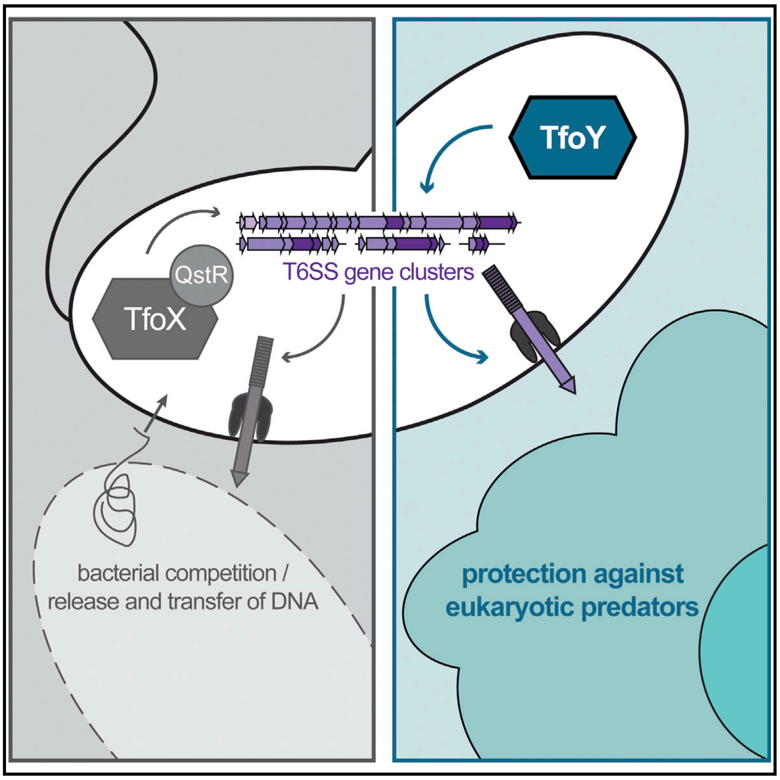

Authors

Lisa C. Metzger, Sandrine Stutzmann, Tiziana Scrignari, Charles Van der Henst, Noémie Matthey, Melanie Blokesch

Correspondence

melanie.blokesch@epfl.ch

In Brief

Metzger et al. find that the type VI secretion system (T6SS) of *Vibrio cholerae* is activated by TfoX and TfoY. Such dual regulation of the T6SS of *V. cholerae* suggests that this molecular killing device is activated by diverse environmental cues.

Highlights

- The type VI secretion system (T6SS) of *V. cholerae* is activated by TfoX and TfoY
- Both regulators aim at different phenotypic outcomes
- TfoY drives the production of T6SS-dependent and T6SS-independent toxins
- The absence of TfoY severely impairs constitutive T6SS activity in strain V52

Accession Numbers

GSE79467

Metzger et al., 2016, Cell Reports *15*, 951–958
May 3, 2016 ©2016 The Authors
http://dx.doi.org/10.1016/j.celrep.2016.03.092

CellPress

Cell Reports
Report

OPEN ACCESS
CellPress

Independent Regulation of Type VI Secretion in *Vibrio cholerae* by TfoX and TfoY

Lisa C. Metzger,[1] Sandrine Stutzmann,[1] Tiziana Scrignari,[1] Charles Van der Henst,[1] Noémie Matthey,[1] and Melanie Blokesch[1,*]
[1]Laboratory of Molecular Microbiology, Global Health Institute, School of Life Sciences, Ecole Polytechnique Fédérale de Lausanne (EPFL), 1015 Lausanne, Switzerland
*Correspondence: melanie.blokesch@epfl.ch
http://dx.doi.org/10.1016/j.celrep.2016.03.092

SUMMARY

Type VI secretion systems (T6SSs) are nanomachines used for interbacterial killing and intoxication of eukaryotes. Although *Vibrio cholerae* is a model organism for structural studies on T6SSs, the underlying regulatory network is less understood. A recent study showed that the T6SS is part of the natural competence regulon in *V. cholerae* and is activated by the regulator TfoX. Here, we identify the TfoX homolog TfoY as a second activator of the T6SS. Importantly, despite inducing the same T6SS core machinery, the overall regulons differ significantly for TfoX and TfoY. We show that TfoY does not contribute to competence induction. Instead, TfoY drives the production of T6SS-dependent and T6SS-independent toxins, together with an increased motility phenotype. Hence, we conclude that *V. cholerae* uses its sole T6SS in response to diverse cues and for distinctive outcomes: either to kill for the prey's DNA, leading to horizontal gene transfer, or as part of a defensive escape reaction.

INTRODUCTION

Vibrio cholerae is a common resident of aquatic habitats and is often found in association with chitinous surfaces (Lipp et al., 2002). Upon growth on chitinous surfaces, *V. cholerae* enters a state of natural competence for transformation (Meibom et al., 2005), which enables the bacterium to take up free DNA through its DNA-uptake machinery (Seitz and Blokesch, 2013; Seitz et al., 2014). Competence regulation in *V. cholerae* involves a complex regulatory network (Metzger and Blokesch, 2016). Briefly, upon growth to high-cell density (HCD; measured by quorum sensing [QS] and the QS regulator HapR; reviewed by Rutherford and Bassler, 2012) on chitin, *V. cholerae* produces the competence activators TfoX and QstR (Lo Scrudato and Blokesch, 2013; Meibom et al., 2005), both of which positively regulate the essential parts of the DNA-uptake machinery (Lo Scrudato and Blokesch, 2012, 2013; Seitz and Blokesch, 2013). We recently demonstrated that the type VI secretion systems (T6SSs) of pandemic *V. cholerae* strains (i.e., the current seventh cholera pandemic) is part of this chitin-induced and TfoX-driven natural competence regulon and leads to the lysis of neighboring non-immune bacteria, followed by the uptake of their genetic material (Borgeaud et al., 2015). The T6SS therefore enhances horizontal gene transfer, as it frees genomic DNA from prey cells (Borgeaud et al., 2015).

T6SSs are present in ~25% of all Gram-negative bacteria. These systems are molecular killing devices used for bacterial warfare and for the intoxication of eukaryotic cells (Ho et al., 2014; Russell et al., 2014). The T6SS consists of two main parts: a membrane-spanning part and a phage-like baseplate structure, to which a tail complex is attached (Costa et al., 2015). The latter is composed of an inner tube made of hemolysin-coregulated (Hcp) proteins, decorated on the outside with a contractile sheath structure (made of VipA and VipB proteins for *V. cholerae*). Upon contraction of the sheath, the Hcp tube and its tip proteins are propelled into neighboring cells (Basler, 2015; Ho et al., 2014). The concomitant delivery of effector toxins leads to the killing of neighboring bacteria or eukaryotic cells. Kin discrimination occurs via the production of effector-compatible immunity proteins that prevent self-destruction (Durand et al., 2014; Russell et al., 2014).

Most studies on the function and structure of the T6SS of *V. cholerae* have been performed in two non-pandemic isolates (V52 and 2740-80) that are constitutively operational with respect to T6SS activity. The rationale behind utilizing these specific strains was that current pandemic *V. cholerae* strains were considered T6SS silent under laboratory conditions (Ho et al., 2014). Indeed, until we reported chitin as an environmental inducer of the system (involving the competence regulator TfoX; Borgeaud et al., 2015), the major trigger that significantly activates T6SS in pandemic strains remained largely unknown (Ho et al., 2014).

Interestingly, *V. cholerae* and other members of the genus *Vibrio* contain an additional TfoX-like protein, designated TfoY (Pollack-Berti et al., 2010) (former name TfoX^{GEMM}; Weinberg et al., 2007). Pollack-Berti et al. (2010) showed that both proteins, TfoX and TfoY, contribute to efficient natural transformation in the symbiotic bacterium *Vibrio fischeri* without being functionally identical. Moreover, these authors suggested differential regulation patterns for *tfoX* and *tfoY* of *V. fischeri*, as their transcriptional activation appeared sequential upon colonization of the light organ of the symbiotic partner (the squid) (Pollack-Berti et al., 2010; Wier et al., 2010). However, regulation of *tfoY* and

This is an open access article under the CC BY license (http://creativecommons.org/licenses/by/4.0/).

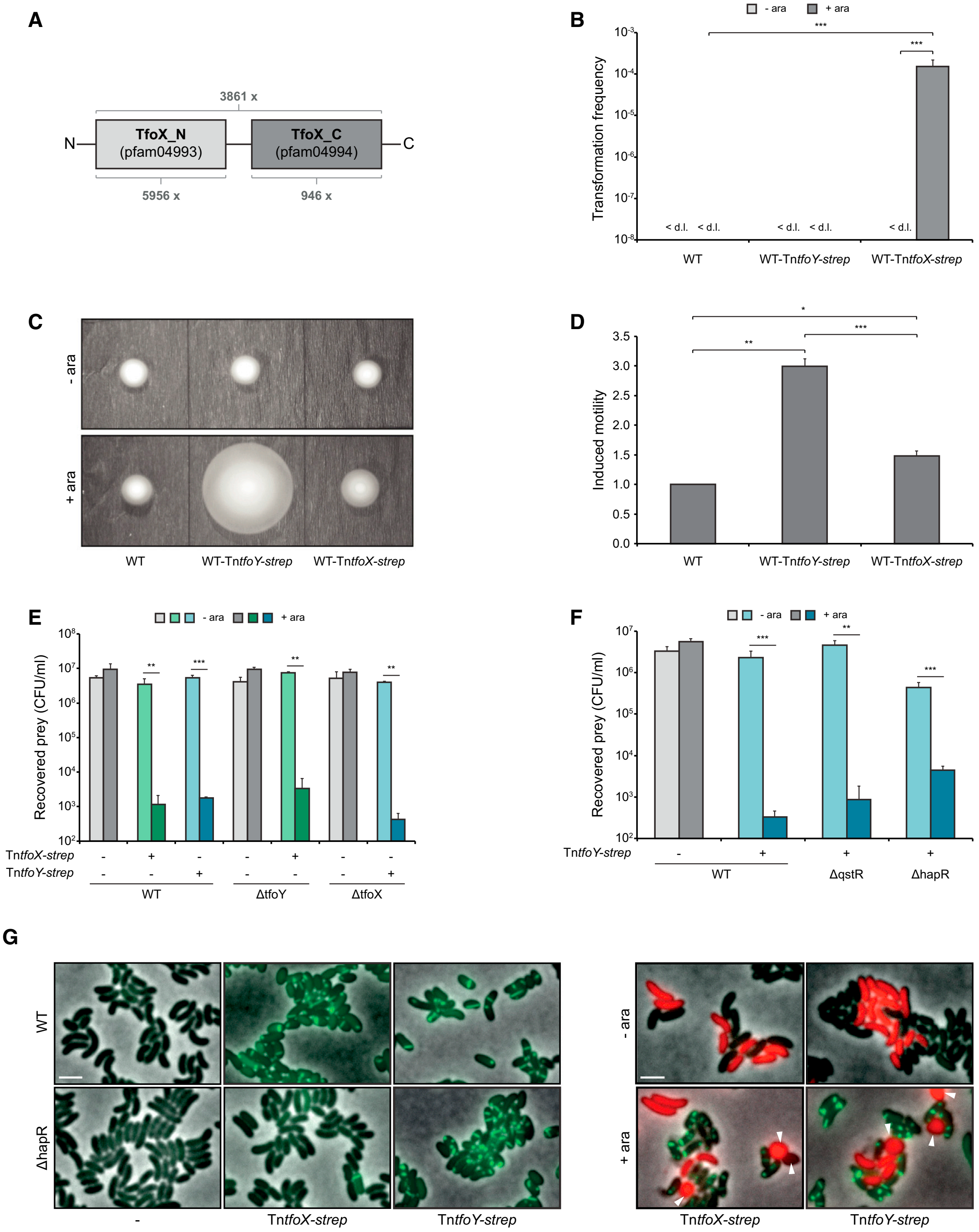

Figure 1. TfoX and TfoY Are Not Redundant

(A) Scheme showing the two-domain structure of TfoX-like proteins. The abundance of proteins carrying either one or both domains is indicated below and above the scheme, respectively.

(B–D) *V. cholerae* strains carrying a chromosomal copy of either *tfoX* or *tfoY* under the control of P_{BAD} (Tn*tfoX-strep* and Tn*tfoY-strep*) were analyzed for chitin-independent natural transformability (B) and motility on semi-solid LB agar (C and D). (B) Natural transformation is fully dependent on TfoX. The indicated strains were cultured under non-inducing (−ara) and inducing (+ara) conditions. Transformation frequencies are shown on the y axis and represent the average of three independent experiments (error bars indicate SD). <d.l., below detection limit. (C and D) TfoY induction vastly enhances surface motility. Motility was scored on soft agar without (−ara) and with (+ara) induction. Representative images are shown (C). (D) Quantification of the motility phenotype shown in (C). The average ratio between induced versus uninduced conditions is shown on the y axis based on three independent experiments (±SD).

(legend continued on next page)

any TfoY-driven transformation-independent phenotypes was not addressed.

TfoX-like proteins are commonly annotated as competence/transformation regulators. Notably, in this study we demonstrate that TfoY of *V. cholerae* does not contribute to natural competence for transformation. Instead, we identified TfoY as a second master regulator of T6SS in *V. cholerae.* T6SS activation by TfoY occurs independently of TfoX, as well as in a chitin- and QS-independent manner. Importantly, we provide evidence that TfoY is not only responsible for T6SS regulation in the most prevalent *V. cholerae* pandemic strains but also for constitutive T6SS activity in the non-pandemic strain V52. Based on comparison between the TfoX and TfoY regulons and the different phenotypes associated with them, we conclude that these two T6SS regulators initiate distinctive cell fates.

RESULTS

The Competence Activator TfoX and Its Homolog TfoY

TfoX is the main activator (together with HapR and QstR) of the natural competence regulon of *V. cholerae*, which includes the T6SS (Borgeaud et al., 2015). TfoX (also referred to as Sxy in other bacteria, such as *Haemophilus influenzae*) (Redfield, 1991) and its homologs consist of two domains (TfoX-N and TfoX-C) according to the protein families database Pfam (Finn et al., 2014) (Figure 1A). Closer inspection of this organization indicated the presence of homologous domains in a plethora of other bacteria (Pfam database [v.28.0]; Finn et al., 2014) (Figure S1), suggesting a pivotal role for these protein domains.

While TfoX-driven phenotypes are well established in *V. cholerae* (Metzger and Blokesch, 2016), TfoY has not previously been studied. We therefore tested whether a *tfoY* mutant of this organism was impaired for chitin-induced natural transformation and found this was not the case (Figure S1C). Likewise, the artificial expression of *tfoY* did not result in natural transformation, in contrast to its TfoX counterpart (Figure 1B).

Although the TfoY-associated phenotypes remain unknown, a c-di-GMP riboswitch within the 5′ UTR of *tfoY* has been identified (Sudarsan et al., 2008). As c-di-GMP is an important second messenger and often involved in the transition from a planktonic (low c-di-GMP) to a sessile (e.g., biofilm at high c-di-GMP) lifestyle (Römling et al., 2013), we tested whether TfoY affected the motility of *V. cholerae*. Indeed, a *tfoY* mutant showed slightly but significantly decreased motility on soft-agar plates (Figure S1D), whereas TfoY overproduction led to a substantial increase in motility (Figure 1). This strong motility phenotype was not reproduced upon TfoX expression (Figure 1), highlighting that the two proteins are truly distinct in function in *V. cholerae*.

TfoY Is a TfoX-Independent Regulator of T6SS

In an attempt to decipher the TfoY regulon and to potentially identify the genes involved in TfoY-mediated motility, we used an RNA-seq approach. Unexpectedly, TfoY induction also induced the T6SS gene clusters of *V. cholerae*, although not to the same extent as TfoX (Borgeaud et al., 2015) (Table S1). We therefore tested whether this TfoY-mediated increase in T6SS gene expression coincided with interbacterial killing and found this was indeed the case (Figure 1E). The killing occurred at high levels comparable to those exerted by the constitutively T6SS-active strain V52 (see below) and at levels equal to the results obtained by TfoX induction (Figure 1E). This TfoY-mediated killing was fully dependent on the presence of the T6SS (Figure S1E). Importantly, neither TfoX nor TfoY required the other to induce T6SS gene expression or to kill neighboring bacteria (Figure 1E).

To test whether TfoY-mediated T6SS induction also required co-regulation by the QS regulator HapR and QstR (Borgeaud et al., 2015), we analyzed different mutants for TfoY-induced interbacterial killing. Interestingly, and again in contrast to TfoX-dependent regulation, the TfoY-expressing mutants that lacked HapR and QstR still proficiently killed their prey (Figure 1F), indicating that QS was not essential for this regulatory circuit.

Next, we visualized the T6SS sheath protein VipA fused to sfGFP (Basler et al., 2012; Borgeaud et al., 2015) upon TfoX or TfoY induction using fluorescence microscopy. As shown in Figure 1G, both regulators were able to drive VipA production and T6SS assembly. This imaging technique also confirmed that while TfoX-mediated T6SS induction required HapR, TfoY-driven T6SS production worked independently of this QS regulator (Figure 1G). Prey rounding and lysis was likewise caused by the induction of TfoX or TfoY (Figure 1G). Altogether, we conclude that both proteins, TfoX and TfoY, induce T6SS through the involvement of distinctive co-regulatory pathways.

VasH Differentially Contributes to TfoX- or TfoY-Mediated T6SS Induction

V. cholerae possesses a single T6SS (Pukatzki et al., 2006), encoded by a large/major gene cluster and two auxiliary clusters. A third auxiliary cluster, which encodes another set of effector/immunity proteins, was recently identified (Altindis et al., 2015) (Figure 2A).

The proteins encoded by the large/major T6SS gene cluster include a putative activator of RpoN (σ^{54}) named VasH (Pukatzki et al., 2006) (Figure 2A). A *vasH* mutant of the *V. cholerae* strain V52 showed a lack of Hcp in the culture supernatant (Kitaoka et al., 2011; Pukatzki et al., 2006), and this highlights the importance of VasH in the constitutively T6SS-active strain (Dong and Mekalanos, 2012; Zheng et al., 2011).

Here, we asked whether and how VasH contributed to TfoX- and TfoY-induced T6SS expression in pandemic *V. cholerae*

(E–G) TfoY induces T6SS-mediated interbacterial killing in a TfoX- and QS-independent manner. (E and F) Interspecies killing assay between *V. cholerae* strains and *E. coli* as prey. Indicated *V. cholerae* were co-cultured with the prey on plain LB agar (−ara) or LB agar plates supplemented with arabinose (+ara) to induce *tfoX* (green) or *tfoY* (blue). The survival of the prey is depicted as colony-forming units (CFU) per ml. Data represent the average of at least three independent biological replicates (±SD). (G) Visualization of T6SS structures (left) and T6SS-induced cell rounding of prey (right) by fluorescence microscopy. Attacked rounded prey cells are indicated by arrowheads. The brightness of the stronger TfoX-induced VipA-sfGFP signal was reduced for better visualization. Statistical significance is indicated for all panels (*p < 0.05; **p < 0.01; ***p < 0.001).
See also Figure S1.

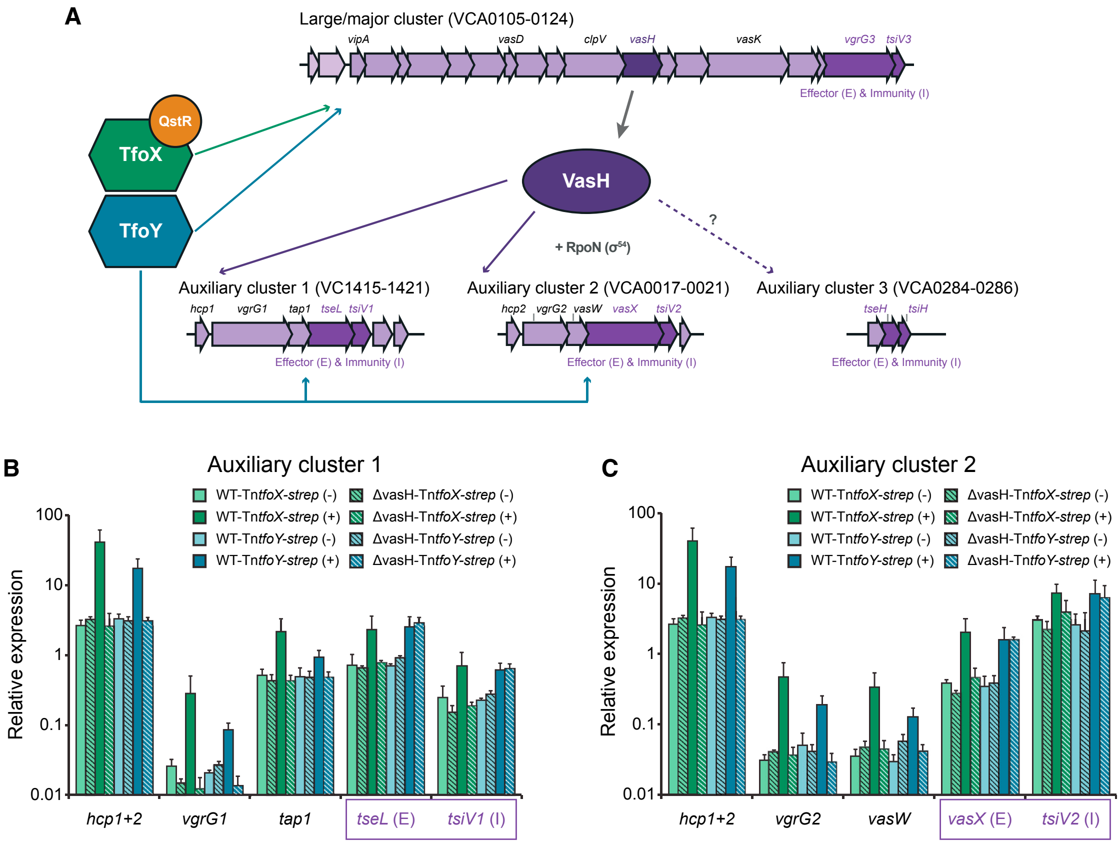

Figure 2. The Regulator VasH Differentially Contributes to TfoX- and TfoY-Mediated T6SS Induction

(A) Scheme describing the TfoX-, TfoY-, and VasH-dependent regulation of the large and auxiliary T6SS gene clusters.

(B and C) The relative expression of genes of the auxiliary T6SS gene clusters 1 (B) and 2 (C) was determined by qRT-PCR. Bacteria were grown in the absence (−) or presence (+) of arabinose to induce *tfoX* (green)/*tfoY* (blue). Data are means of at least three independent biological replicates (±SD). Effector (E)/immunity (I) genes are highlighted by boxes.

See also Figure S2.

strains. Indeed, the protein was essential for both pathways: a *vasH* deletion strain was fully impaired for interbacterial killing upon TfoX or TfoY production (Figures S2A and S2B), even though co-regulated phenotypes, such as competence and motility, were not affected (Figures S2C and S2D). Next, we compared the expression pattern of representative genes of the four T6SS gene clusters. As shown in Figure S2E for the large T6SS cluster, VasH was dispensable for the TfoX- and TfoY-mediated expression (at least for the genes upstream of *vasH*). Moreover, with respect to auxiliary clusters 1 and 2, VasH was essential for the TfoX/TfoY-driven induction of the two *hcp* copies (Figure 2), which we also demonstrated at the protein level (Figure S2F). Likewise, VasH dependency was observed for the downstream genes of *hcp* in both auxiliary clusters upon TfoX/TfoY induction (Figure 2). However, for both clusters, the T6SS effector and immunity-encoding genes (*tseL* & *tsiV1* and *vasX* & *tsiV2*) proved to be VasH dependent only in the case of TfoX induction, while their expression was VasH independent when the system was driven by TfoY (Figure 2). Importantly, the TfoY-mediated induction of these effector-encoding genes not only occurred independently of VasH but also without a need for RpoN (Figure S2G). Finally, the recently identified effector-immunity gene pair within the third auxiliary cluster (Altindis et al., 2015) was not inducible by TfoY and was only statistically insignificantly inducible by TfoX (Figure S2H). Consistent with these expression data was the finding that TfoX- and TfoY-initiated interbacterial killing did not differ between the wild-type (WT) and a *tseH tsiH* knockout strain (Figure S2I). Hence, this T6SS effector, which most likely exhibits peptidoglycan hydrolase activity (Altindis et al., 2015), seems not to contribute to TfoX- and TfoY-induced T6SS-mediated phenotypes under the tested conditions.

Absence of TfoY Significantly Impairs the Constitutive T6SS Activity in Strain V52

The non-pandemic strain V52 efficiently kills other Gram-negative bacteria due to its constitutive T6SS activity (MacIntyre et al., 2010) and despite a mutation that results in a premature stop codon within *hapR* (Chun et al., 2009), which renders the strain unable to enter natural competence (data not shown). We therefore wondered whether there was a link between constitutive T6SS production and the QS-independent T6SS

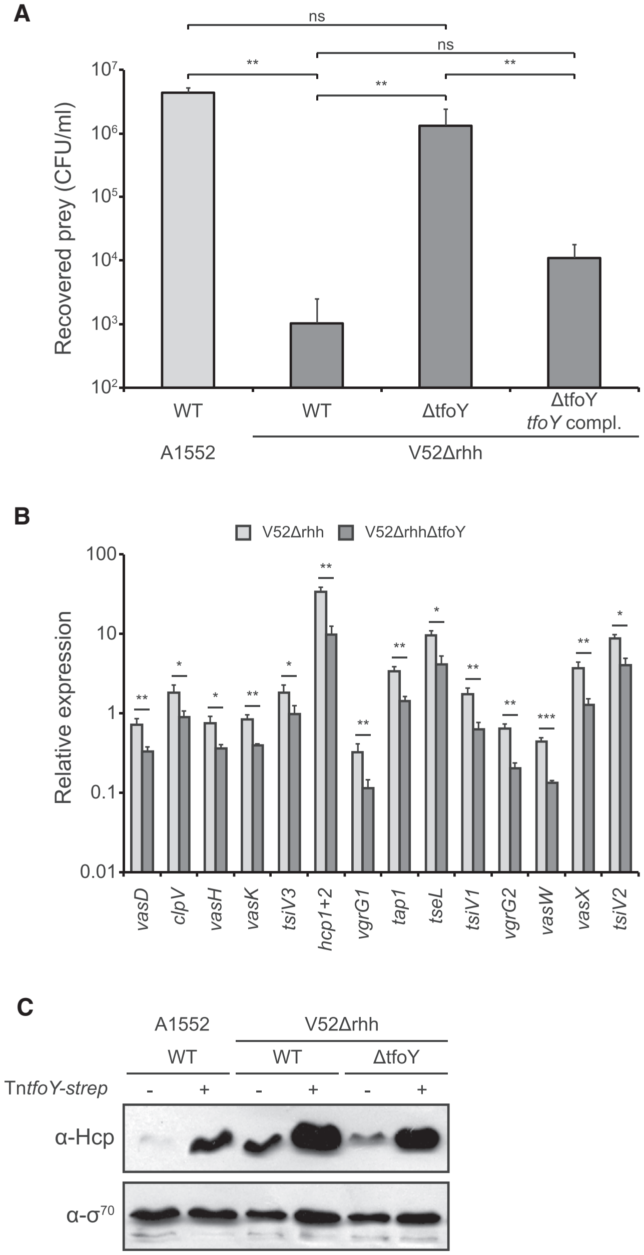

Figure 3. TfoY Is Primarily Responsible for Constitutive T6SS Expression in Strain V52

(A) An interbacterial killing assay with the indicated V52-derived predator strains. *E. coli* prey and the indicated predator were co-cultured on LB agar plates containing arabinose. ΔtfoY *tfoY* compl., ΔtfoY strain carrying complementing Tn*tfoY*-strep on the chromosome. Details are as in Figure 1.

(B) Relative gene expression of representative T6SS genes comparing the *tfoY*-minus (V52ΔrhhΔtfoY) mutant to its parental strain (V52Δrhh).

(C) Hcp production is reduced in the absence of TfoY in strain V52. Proteins of the indicated bacterial strains were detected by western blotting using Hcp-specific antibodies. The absence or presence of inducible *tfoY* (Tn*tfoY*-strep) is shown above the image. All strains were grown in the presence of the inducer (ara). Detection of σ^{70} served as the loading control. Statistical values are indicated (ns, not significant; *$p < 0.05$; **$p < 0.01$; ***$p < 0.001$).

See also Figure S3.

regulator TfoY. Indeed, upon deletion of *tfoY* in strain V52, T6SS-mediated killing was almost abrogated (Figures 3A and S3A). In contrast and as expected, a deletion of *tfoX* did not interfere with T6SS-mediated killing by strain V52 (recovered prey was $7.4 \times 10^3 \pm 9.2 \times 10^3$ for the *tfoX* mutant compared to $4.0 \times 10^3 \pm 3.9 \times 10^3$ for the parental strain; average of three independent experiments ± SD). Moreover, *tfoY* deletion in strain V52 resulted in a significant decrease in T6SS gene expression as determined by qRT-PCR (Figures 3B and S3B) and accordingly in a decrease in the Hcp protein level (Figure 3C). Notably, both interbacterial killing and Hcp production could be restored in the *tfoY*-minus V52 mutant strain by re-introduction of the inducible copy of *tfoY* (Figure 3). Lastly, the lack of TfoY also significantly impaired amoebal killing (Table S2), as determined by a *Dictyostelium discoideum* plaque formation assay (Pukatzki et al., 2006). In this assay, strain V52 reduced the amoebal population to 10%, whereas the *tfoY*-minus derivative of the strain resulted in ~95% survival (compared to a *Klebsiella*-negative control; Table S2). We therefore conclude that TfoY has a major impact on the constitutive T6SS activity in this well-studied *V. cholerae* strain V52.

Role of c-di-GMP in TfoY Production and TfoY-Mediated Processes

We provided a first hint regarding the TfoY regulon and TfoY-associated phenotypes, but the question of how TfoY is regulated remained. As noted above, Sudarsan et al. (2008) identified a c-di-GMP riboswitch upstream *tfoY*, suggesting the involvement of the second messenger c-di-GMP in TfoY production. To elucidate the potential effect of intracellular c-di-GMP levels on TfoY activity, we engineered *V. cholerae* strains carrying an inducible copy of a c-di-GMP-producing diguanylate cyclase-encoding gene (*vdcA*) or a phosphodiesterase-encoding gene (*cdpA*) on the chromosome. The activities of the two enzymes have previously been characterized (Tamayo et al., 2008). These authors demonstrated an increase and decrease of c-di-GMP upon expression of VdcA and CdpA, respectively. When we tested these *vdcA-/cdpA*-inducible strains in a motility assay, we observed, as expected, reduced motility upon c-di-GMP increase, while degradation of the second messenger vastly enhanced motility (Figure S4A). Notably, the presence or absence of *tfoY* did not influence this outcome, indicating that TfoY is not essential for the high-motility phenotype observed under low c-di-GMP conditions.

Next, we wondered whether a change in intracellular c-di-GMP levels would interfere with bacterial killing and therefore tested the effect of the above-mentioned c-di-GMP-variable strains on co-cultured *Escherichia coli*. For this approach, we used a WT strain carrying a functional TfoY-mCherry translational fusion-encoding allele (Figure S4B) at the gene's native locus (Table S3). As indicated in Figure S4C, a significant reduction in *E. coli* recovery was repeatedly observed upon expression of *cdpA*, which was fully dependent on the presence of *tfoY* (Figure S4D). Consistent with this finding was the detection of the TfoY-mCherry protein by western blotting upon induction of the phosphodiesterase (Figure S4E), which suggests that TfoY production is (at least in part) triggered by low c-di-GMP levels.

OPEN ACCESS
CellPress

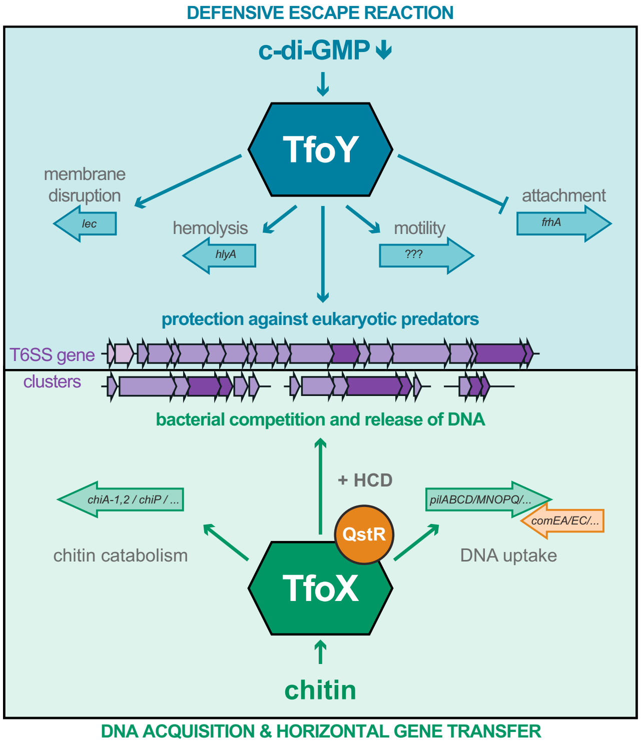

Figure 4. TfoY Induces a Defensive Escape Reaction

Working model differentiating TfoX- and TfoY-mediated responses in *V. cholerae*. For details, see text.

See also Figure S4.

DISCUSSION

The regulatory network that drives T6SS expression in the well-studied seventh pandemic *V. cholerae* isolates has been sorely neglected in the past. Here, we addressed this lack of knowledge and demonstrated that the two regulatory proteins TfoX and TfoY significantly induce the T6SS, leading to highly efficient interbacterial killing, comparable to the killing observed in the non-pandemic constitutively T6SS-active strain V52. It was known that TfoX is produced upon growth on chitin (Meibom et al., 2005), but the production and function of TfoY remained unknown for *V. cholerae*. Based on a riboswitch associated with *tfoY* (Sudarsan et al., 2008), we tested the contribution of the secondary messenger c-di-GMP to the production of TfoY. Our data showed that decreased c-di-GMP levels enforce the production of the TfoY protein and associated phenotypes (e.g., T6SS-mediated killing). Notably, as we only observed a small but significant effect on T6SS-mediated killing (Figure S4), we hypothesize that additional signals are required for full TfoY production in nature. Indeed, a combination of transcriptional and translational control for TfoY production, similar to what has been described for TfoX (reviewed by Metzger and Blokesch, 2016), seems likely and will be addressed in future work. This impact of c-di-GMP on T6SS activity in *V. cholerae* was unexpected and opposite of that described for *Pseudomonas aeruginosa.* Indeed, in *P. aeruginosa* the H1-T6SS is produced at high c-di-GMP levels, concomitant with enhanced biofilm formation (Moscoso et al., 2011).

With respect to the expression of the T6SS genes, Ho et al. (2014) speculated “that RpoN and VasH control only the *hcp* operons and not the main cluster suggests a two-tiered regulatory cascade. Environmental signals first need to trigger the transcription of the major cluster so that *vasH* is expressed, which subsequently activates the transcription of the *hcp* operons by RpoN.” Here, we provide evidence regarding the initial input into this two-tiered regulatory cascade. TfoX and TfoY are produced, respectively, upon reaching HCD on chitinous surfaces and through the reduction of intracellular c-di-GMP levels (Figure 4). These two regulators both initiate expression of the large T6SS cluster, including *vasH*. VasH production subsequently leads to expression of the auxiliary clusters 1 and 2 (Figure 2A). An important exception to this general regulation occurred, however: in the absence of VasH, the effector/immunity encoding genes of the auxiliary clusters 1 and 2 were still induced in a TfoY-dependent manner (Figure 2) that was not observed upon TfoX induction. We hypothesize that this superior activation by TfoY might be explained by the biological function of these two encoded effector proteins. TseL and VasX possess lipase and pore-forming activity (Dong et al., 2013; Miyata et al., 2013; Russell et al., 2013), respectively, both of which are not only functional against prokaryotes but importantly also against eukaryotes. Indeed, it has been previously shown that TseL and VasX are required to fight predation by *D. discoideum* in the T6SS-hyperactive strain V52 (Dong et al., 2013). Thus, we hypothesize that the TfoY-mediated response aims at targeting eukaryotic predators. Consistent with this idea, we demonstrated that *V. cholerae* strain V52 is severely impaired for amoebal killing in the absence of *tfoY.* In addition, a TfoY-mediated defense reaction is supported by the changed expression of several other genes, as elucidated by RNA-seq. For instance, TfoY represses the “flagellum-regulated hemagglutinin A” gene (*frhA*; 3.5-fold repression), a bacterial adhesin required for attachment (Syed et al., 2009). This detachment phenotype accompanies the enhanced motility observed upon TfoY induction (Figure 1). Moreover, TfoY led to a strong induction of hemolysin (*hlyA*; Alm et al., 1988; 8.4-fold induction) and of a lecithinase (*lec*; Fiore et al., 1997; also known as thermolabile hemolysin, *tlh*; 10-fold induction), both of which also target eukaryotic cells. Accordingly, the corresponding activities were strongly reduced in a *tfoY* mutant compared to the WT (Figure S4).

In summary, we conclude that *V. cholerae* uses two independent regulatory pathways to induce its single T6SS: first, to kill for DNA as part of its natural competence program (Borgeaud et al., 2015), and second, to kill as part of a defensive escape reaction (Figure 4). These two responses are driven by TfoX and TfoY, respectively, and provide the bacterium with the unique ability to use the same T6SS for different purposes. Further studies will show whether potential danger sensing (LeRoux et al., 2015b) contributes to full TfoY production and whether the TfoY-mediated phenotypes described in this study aim at fighting a potential threat, as previously suggested for *P. aeruginosa* (LeRoux et al., 2015a).

EXPERIMENTAL PROCEDURES

Bacterial Strains, Plasmids, and Growth Conditions

The *V. cholerae* strains and plasmids used in this study are listed in Table S3. Detailed information on growth conditions and plasmid/strain constructions is provided in the Supplemental Experimental Procedures.

Interbacterial Killing Assay

The interbacterial killing assay was performed as previously described (Borgeaud et al., 2015). TOP10 (Invitrogen), TOP10-TnKan (this study), and SM10λpir (Simon et al., 1983) were used as *E. coli* prey and competitors.

RNA Sequencing and Data Analysis

Bacterial growth, RNA preparation, and DNase treatment, as well as ribodepletion, library preparation, RNA sequencing (RNA-seq), and data analysis (Microsynth) were performed as previously described (Borgeaud et al., 2015). The TfoX-induced dataset is derived from a previous study (Borgeaud et al., 2015), whereas the WT control (A1552) and the TfoY-induced dataset are part of this study (GEO: GSE79467).

Gene Expression Analysis by qRT-PCR

qRT-PCR-based analysis of gene expression in *V. cholerae* was performed as previously described, and the relative expression values are based on normalization against *gyrA* (Lo Scrudato and Blokesch, 2012).

SDS-PAGE and Western Blotting

Proteins from bacterial cell lysates were separated by SDS-PAGE and subjected to western blotting as previously described (Lo Scrudato and Blokesch, 2012). Specific proteins were detected using α-Hcp (Eurogentec), α-σ^{70} (BioLegend), and α-mCherry (BioVision) as the primary antibodies.

D. discoideum Plaque Assay

To determine the cytotoxicity of the *V. cholerae* strain V52 and its *tfoY*-minus derivative toward the amoeba *D. discoideum* (strain DH1), a plaque assay was performed similar to the one previously described (Pukatzki et al., 2006) (see the Supplemental Experimental Procedures).

ACCESSION NUMBERS

The accession number for the WT control (A1552) and the TfoY-induced dataset reported in this paper is GEO: GSE79467.

SUPPLEMENTAL INFORMATION

Supplemental Information includes Supplemental Experimental Procedures, four figures, and three tables and can be found with this article online at http://dx.doi.org/10.1016/j.celrep.2016.03.092.

AUTHOR CONTRIBUTIONS

L.C.M. and M.B. designed the experiments. L.C.M., S.S., T.S., C.V.d.H., and M.B. performed the experiments. C.V.d.H. and N.M. contributed strains and ideas. L.C.M. and M.B. analyzed the data. L.C.M. and M.B. wrote the paper.

ACKNOWLEDGMENTS

We thank all the researchers who provided us with bacterial and amoebal strains. This work was supported by the Swiss National Science Foundation (31003A_143356 and 31003A_162551) and the European Research Council (309064-VIR4ENV).

Received: December 21, 2015
Revised: February 16, 2016
Accepted: March 27, 2016
Published: April 21, 2016

REFERENCES

Alm, R.A., Stroeher, U.H., and Manning, P.A. (1988). Extracellular proteins of *Vibrio cholerae*: nucleotide sequence of the structural gene (*hlyA*) for the haemolysin of the haemolytic El Tor strain O17 and characterization of the *hlyA* mutation in the non-haemolytic classical strain 569B. Mol. Microbiol. *2*, 481–488.

Altindis, E., Dong, T., Catalano, C., and Mekalanos, J. (2015). Secretome analysis of *Vibrio cholerae* type VI secretion system reveals a new effector-immunity pair. mBio *6*, e00075-15.

Basler, M. (2015). Type VI secretion system: secretion by a contractile nanomachine. Philos. Trans. R. Soc. Lond. B. Biol. Sci. *370*, pii: 20150021.

Basler, M., Pilhofer, M., Henderson, G.P., Jensen, G.J., and Mekalanos, J.J. (2012). Type VI secretion requires a dynamic contractile phage tail-like structure. Nature *483*, 182–186.

Borgeaud, S., Metzger, L.C., Scrignari, T., and Blokesch, M. (2015). The type VI secretion system of *Vibrio cholerae* fosters horizontal gene transfer. Science *347*, 63–67.

Chun, J., Grim, C.J., Hasan, N.A., Lee, J.H., Choi, S.Y., Haley, B.J., Taviani, E., Jeon, Y.S., Kim, D.W., Lee, J.H., et al. (2009). Comparative genomics reveals mechanism for short-term and long-term clonal transitions in pandemic *Vibrio cholerae*. Proc. Natl. Acad. Sci. USA *106*, 15442–15447.

Costa, T.R.D., Felisberto-Rodrigues, C., Meir, A., Prevost, M.S., Redzej, A., Trokter, M., and Waksman, G. (2015). Secretion systems in Gram-negative bacteria: structural and mechanistic insights. Nat. Rev. Microbiol. *13*, 343–359.

Dong, T.G., and Mekalanos, J.J. (2012). Characterization of the RpoN regulon reveals differential regulation of T6SS and new flagellar operons in *Vibrio cholerae* O37 strain V52. Nucleic Acids Res. *40*, 7766–7775.

Dong, T.G., Ho, B.T., Yoder-Himes, D.R., and Mekalanos, J.J. (2013). Identification of T6SS-dependent effector and immunity proteins by Tn-seq in *Vibrio cholerae*. Proc. Natl. Acad. Sci. USA *110*, 2623–2628.

Durand, E., Cambillau, C., Cascales, E., and Journet, L. (2014). VgrG, Tae, Tle, and beyond: the versatile arsenal of Type VI secretion effectors. Trends Microbiol. *22*, 498–507.

Finn, R.D., Bateman, A., Clements, J., Coggill, P., Eberhardt, R.Y., Eddy, S.R., Heger, A., Hetherington, K., Holm, L., Mistry, J., et al. (2014). Pfam: the protein families database. Nucleic Acids Res. *42*, D222–D230.

Fiore, A.E., Michalski, J.M., Russell, R.G., Sears, C.L., and Kaper, J.B. (1997). Cloning, characterization, and chromosomal mapping of a phospholipase (lecithinase) produced by *Vibrio cholerae*. Infect. Immun. *65*, 3112–3117.

Ho, B.T., Dong, T.G., and Mekalanos, J.J. (2014). A view to a kill: the bacterial type VI secretion system. Cell Host Microbe *15*, 9–21.

Kitaoka, M., Miyata, S.T., Brooks, T.M., Unterweger, D., and Pukatzki, S. (2011). VasH is a transcriptional regulator of the type VI secretion system functional in endemic and pandemic *Vibrio cholerae*. J. Bacteriol. *193*, 6471–6482.

LeRoux, M., Kirkpatrick, R.L., Montauti, E.I., Tran, B.Q., Peterson, S.B., Harding, B.N., Whitney, J.C., Russell, A.B., Traxler, B., Goo, Y.A., et al. (2015a). Kin cell lysis is a danger signal that activates antibacterial pathways of *Pseudomonas aeruginosa*. eLife *4*, e05701.

LeRoux, M., Peterson, S.B., and Mougous, J.D. (2015b). Bacterial danger sensing. J. Mol. Biol. *427*, 3744–3753.

Lipp, E.K., Huq, A., and Colwell, R.R. (2002). Effects of global climate on infectious disease: the cholera model. Clin. Microbiol. Rev. *15*, 757–770.

Lo Scrudato, M., and Blokesch, M. (2012). The regulatory network of natural competence and transformation of *Vibrio cholerae*. PLoS Genet. *8*, e1002778.

Lo Scrudato, M., and Blokesch, M. (2013). A transcriptional regulator linking quorum sensing and chitin induction to render *Vibrio cholerae* naturally transformable. Nucleic Acids Res. *41*, 3644–3658.

MacIntyre, D.L., Miyata, S.T., Kitaoka, M., and Pukatzki, S. (2010). The *Vibrio cholerae* type VI secretion system displays antimicrobial properties. Proc. Natl. Acad. Sci. USA *107*, 19520–19524.

Meibom, K.L., Blokesch, M., Dolganov, N.A., Wu, C.-Y., and Schoolnik, G.K. (2005). Chitin induces natural competence in *Vibrio cholerae*. Science *310*, 1824–1827.

Metzger, L.C., and Blokesch, M. (2016). Regulation of competence-mediated horizontal gene transfer in the natural habitat of *Vibrio cholerae*. Curr. Opin. Microbiol. *30*, 1–7.

Miyata, S.T., Unterweger, D., Rudko, S.P., and Pukatzki, S. (2013). Dual expression profile of type VI secretion system immunity genes protects pandemic *Vibrio cholerae*. PLoS Pathog. *9*, e1003752.

Moscoso, J.A., Mikkelsen, H., Heeb, S., Williams, P., and Filloux, A. (2011). The *Pseudomonas aeruginosa* sensor RetS switches type III and type VI secretion via c-di-GMP signalling. Environ. Microbiol. *13*, 3128–3138.

Pollack-Berti, A., Wollenberg, M.S., and Ruby, E.G. (2010). Natural transformation of *Vibrio fischeri* requires *tfoX* and *tfoY*. Environ. Microbiol. *12*, 2302–2311.

Pukatzki, S., Ma, A.T., Sturtevant, D., Krastins, B., Sarracino, D., Nelson, W.C., Heidelberg, J.F., and Mekalanos, J.J. (2006). Identification of a conserved bacterial protein secretion system in *Vibrio cholerae* using the *Dictyostelium* host model system. Proc. Natl. Acad. Sci. USA *103*, 1528–1533.

Redfield, R.J. (1991). sxy-1, a *Haemophilus influenzae* mutation causing greatly enhanced spontaneous competence. J. Bacteriol. *173*, 5612–5618.

Römling, U., Galperin, M.Y., and Gomelsky, M. (2013). Cyclic di-GMP: the first 25 years of a universal bacterial second messenger. Microbiol. Mol. Biol. Rev. *77*, 1–52.

Russell, A.B., LeRoux, M., Hathazi, K., Agnello, D.M., Ishikawa, T., Wiggins, P.A., Wai, S.N., and Mougous, J.D. (2013). Diverse type VI secretion phospholipases are functionally plastic antibacterial effectors. Nature *496*, 508–512.

Russell, A.B., Peterson, S.B., and Mougous, J.D. (2014). Type VI secretion system effectors: poisons with a purpose. Nat. Rev. Microbiol. *12*, 137–148.

Rutherford, S.T., and Bassler, B.L. (2012). Bacterial quorum sensing: its role in virulence and possibilities for its control. Cold Spring Harb. Perspect. Med. *2*, a012427.

Seitz, P., and Blokesch, M. (2013). DNA-uptake machinery of naturally competent *Vibrio cholerae*. Proc. Natl. Acad. Sci. USA *110*, 17987–17992.

Seitz, P., Pezeshgi Modarres, H., Borgeaud, S., Bulushev, R.D., Steinbock, L.J., Radenovic, A., Dal Peraro, M., and Blokesch, M. (2014). ComEA is essential for the transfer of external DNA into the periplasm in naturally transformable *Vibrio cholerae* cells. PLoS Genet. *10*, e1004066.

Simon, R., Priefer, U., and Pühler, A. (1983). A broad host range mobilization system for in vivo genetic engineering: transposon mutagenesis in Gram negative bacteria. Nat. Biotechnol. *1*, 784–791.

Sudarsan, N., Lee, E.R., Weinberg, Z., Moy, R.H., Kim, J.N., Link, K.H., and Breaker, R.R. (2008). Riboswitches in eubacteria sense the second messenger cyclic di-GMP. Science *321*, 411–413.

Syed, K.A., Beyhan, S., Correa, N., Queen, J., Liu, J., Peng, F., Satchell, K.J., Yildiz, F., and Klose, K.E. (2009). The *Vibrio cholerae* flagellar regulatory hierarchy controls expression of virulence factors. J. Bacteriol. *191*, 6555–6570.

Tamayo, R., Schild, S., Pratt, J.T., and Camilli, A. (2008). Role of cyclic Di-GMP during el tor biotype *Vibrio cholerae* infection: characterization of the in vivo-induced cyclic Di-GMP phosphodiesterase CdpA. Infect. Immun. *76*, 1617–1627.

Weinberg, Z., Barrick, J.E., Yao, Z., Roth, A., Kim, J.N., Gore, J., Wang, J.X., Lee, E.R., Block, K.F., Sudarsan, N., et al. (2007). Identification of 22 candidate structured RNAs in bacteria using the CMfinder comparative genomics pipeline. Nucleic Acids Res. *35*, 4809–4819.

Wier, A.M., Nyholm, S.V., Mandel, M.J., Massengo-Tiassé, R.P., Schaefer, A.L., Koroleva, I., Splinter-Bondurant, S., Brown, B., Manzella, L., Snir, E., et al. (2010). Transcriptional patterns in both host and bacterium underlie a daily rhythm of anatomical and metabolic change in a beneficial symbiosis. Proc. Natl. Acad. Sci. USA *107*, 2259–2264.

Zheng, J., Ho, B., and Mekalanos, J.J. (2011). Genetic analysis of anti-amoebae and anti-bacterial activities of the type VI secretion system in *Vibrio cholerae*. PLoS ONE *6*, e23876.

Cell Reports, Volume *15*

Supplemental Information

Independent Regulation of Type VI Secretion in *Vibrio cholerae* by TfoX and TfoY

Lisa C. Metzger, Sandrine Stutzmann, Tiziana Scrignari, Charles Van der Henst, Noémie Matthey, and Melanie Blokesch

SUPPLEMENTAL FIGURES AND LEGENDS

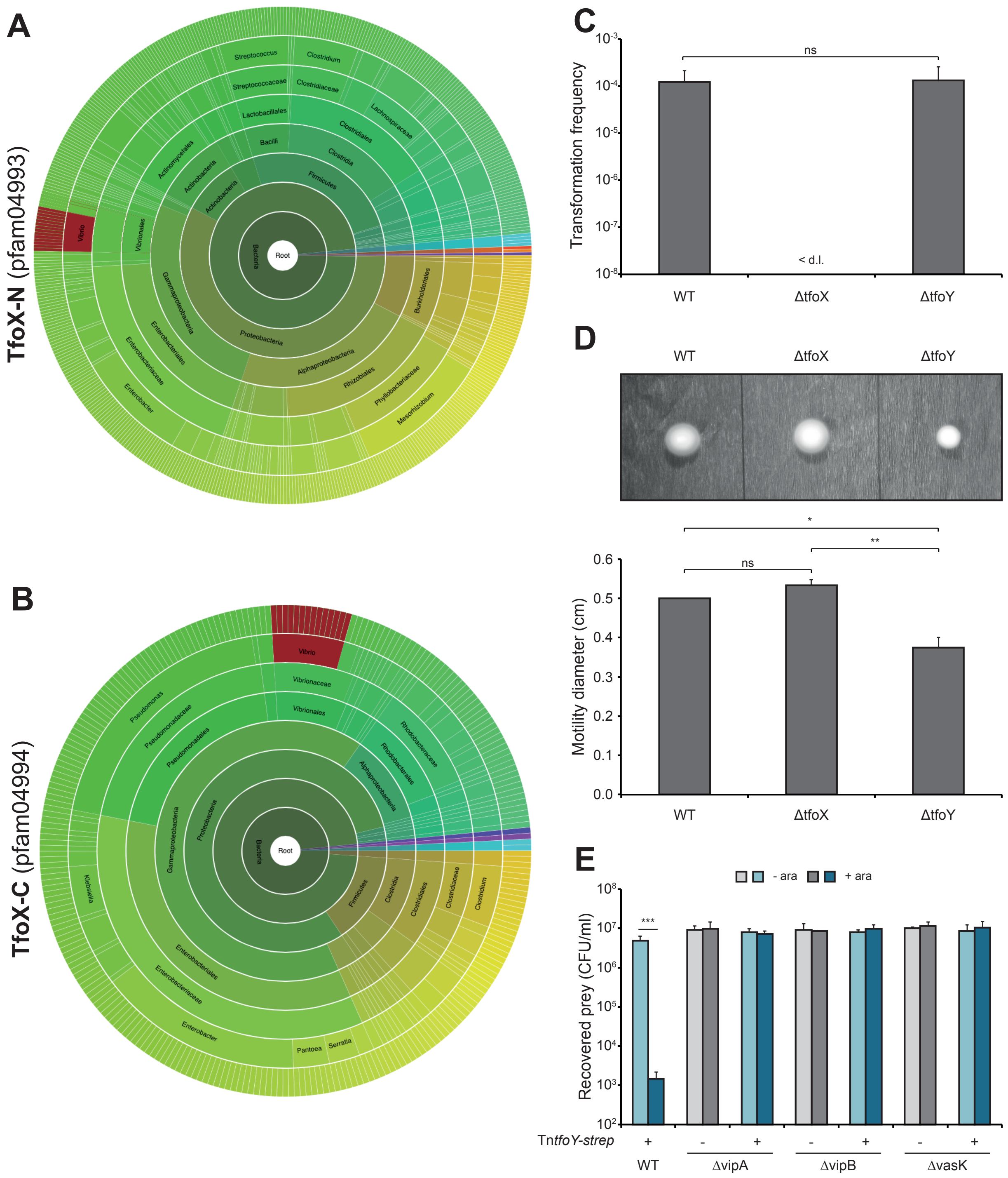

Figure S1: Kingdom-wide distribution of TfoX-like proteins, related to Figure 1. (A and B) Sunburst diagram showing the distribution of the two TfoX-like domains among different species. N-terminal domain of TfoX refers to PF04993 and the C-terminal domain to PF04994. Diagram derived from pfam.xfam.org (Pfam version 28.0). The segments are weighted by number of species. *Vibrio* species are highlighted in red. (C) TfoY is dispensable for chitin-induced natural transformation. Comparison of WT, ΔtfoX, and ΔtfoY strains with respect to natural transformability on chitin. Data are average transformation frequencies of at least three biological replicates (±SD). (D) TfoY influences bacterial motility. Comparison of WT, ΔtfoX, and ΔtfoY strain with respect to motility on soft agar. (E) Quantification of *E. coli* prey cells after co-incubation with the indicated *V. cholerae* predator cells (without (-ara) or with (+ara) arabinose). Details as in Figure 1.

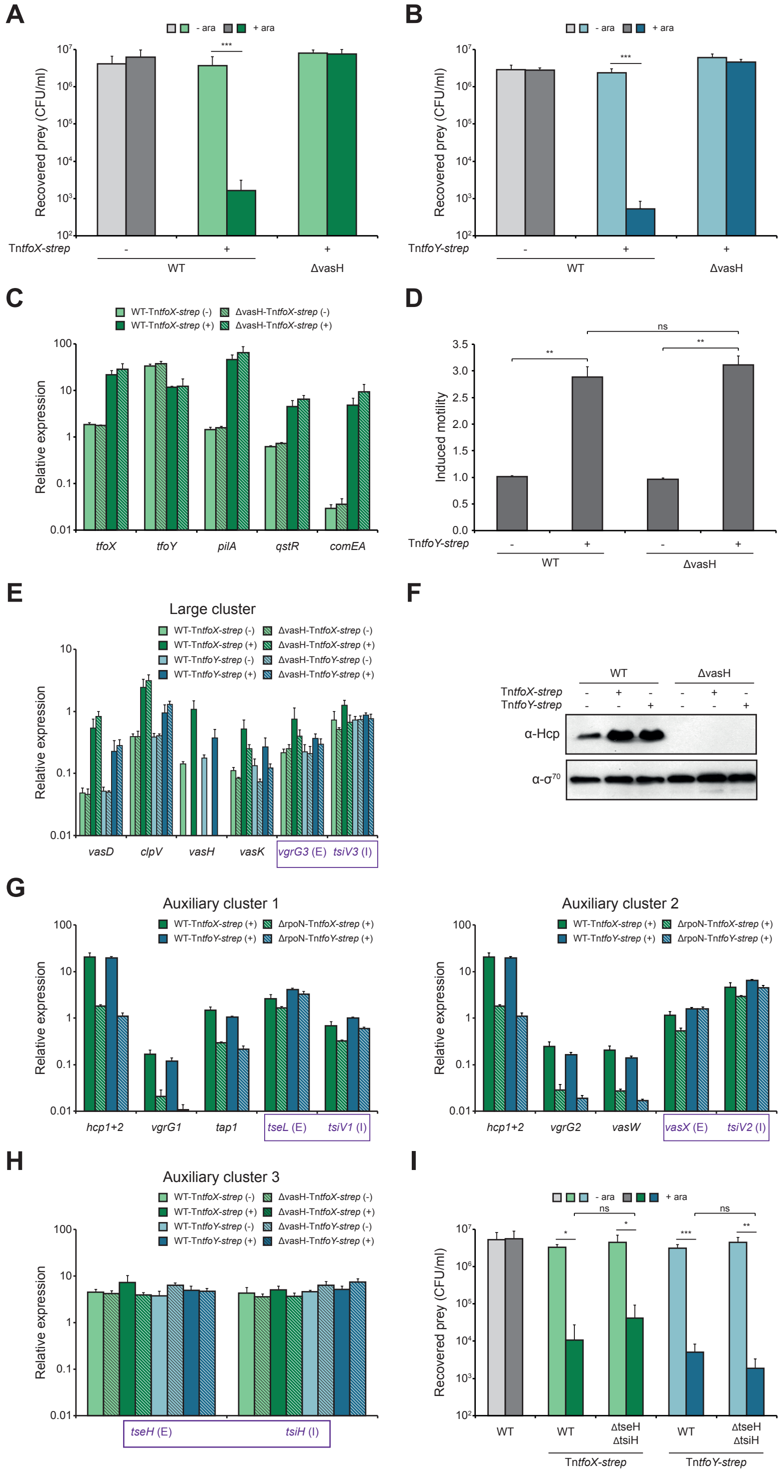

Figure S2: The contribution of VasH to TfoX- and TfoY-dependent T6SS activity, related to Figure 2. (A, B, and I) Interspecies killing assay between indicated *V. cholerae* as predator strains and *E. coli* prey. Quantification of surviving prey cells after the co-culturing with predator as described (see Figure 1). (C) TfoX-mediated competence induction does not rely on VasH. Transcript analysis of representative competence genes (*pilA, qstR, comEA*) in *V. cholerae* WT and a *vasH*-minus strain under *tfoX*-non-inducing (-) and *tfoX*-inducing (+) conditions. (D) TfoY-mediated motility enhancement occurs independently of VasH. Motility was scored for the indicated *V. cholerae* strains (as in Figure 1). (E) Transcript analysis in *V. cholerae* WT & ΔvasH. Relative gene expression analyzed by qRT-PCR as in Figure 2 but for the large T6SS cluster. (F) Detection of Hcp protein by western blotting. Cell lysates from the indicated *V. cholerae* strains grown to HCD (+ara) were analyzed for Hcp abundance. (G) Relative gene expression for the auxiliary clusters 1 and 2 comparing WT and an *rpoN*-minus strain. (H) Transcript analysis of the effector (E) and immunity (I) genes of auxiliary cluster 3. All experiments were performed at least three independent times, and error bars indicate standard deviations.

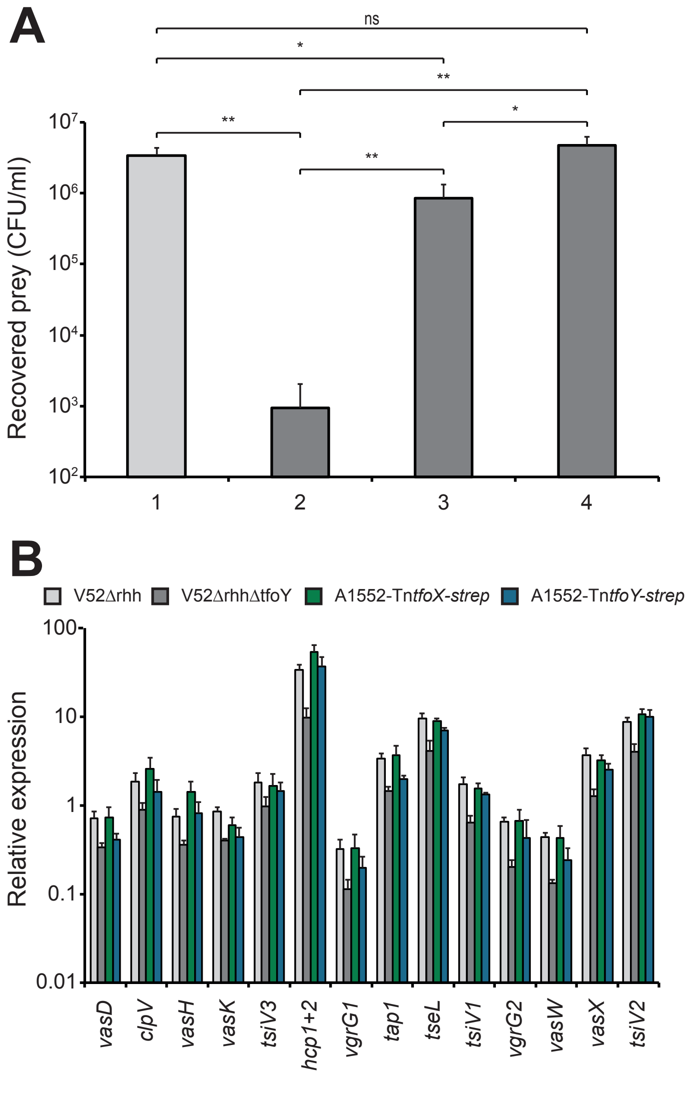

Figure S3: The constitutive T6SS activity of strain V52 depends primarily on TfoY, related to Figure 3. (A) A *tfoY*-minus mutant of V52 is severely impaired for interbacterial killing. Predator and prey were co-incubated on plain LB agar plates. *V. cholerae* predator strains tested (from 1 to 4 on X-axis): A1552, V52Δrhh, V52ΔrhhΔtfoY, and V52ΔvipB (T6SS-minus). Quantification of prey survival as described in Figure 1. (B) Relative expression of selected target genes as quantified by qRT-PCR in *V. cholerae* strains V52Δrhh, V52ΔrhhΔtfoY, A1552-Tn*tfoX*-strep and A1552-Tn*tfoY*-strep. Cells were grown to HCD in the presence of arabinose. Data are averages of three independent experiments (±SD).

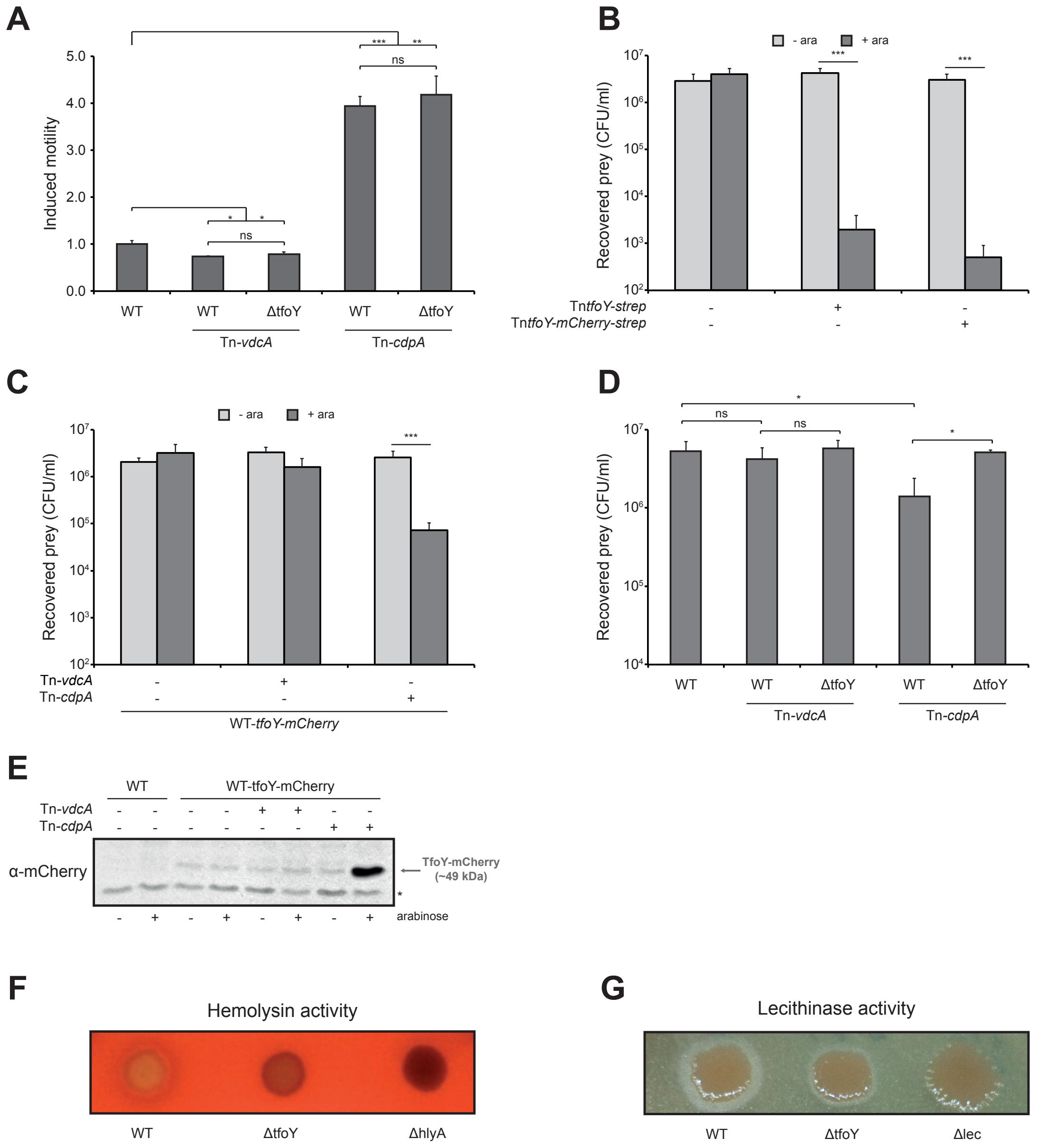

Figure S4: TfoY-mediated phenotypes and the contribution of low c-di-GMP levels to interbacterial killing, related to Figure 4. (A) Lowering the c-di-GMP levels within the cells increases bacterial motility. The indicated *V. cholerae* strains were spotted onto motility agar supplemented with arabinose to induce *vdcA* (a diguanylate cyclase) or *cdpA* (a phosphodiesterase) where indicated (Tn-*vdcA* and Tn-*cpdA*). Motility induction is indicated on the Y-axis. (B) The translational fusion between TfoY and mCherry remains full functionality. Interspecies killing assay between a WT, TfoY- (Tn*tfoY-strep*), and TfoY-mCherry-expressing (Tn*tfoY-mCherry-strep*) *V. cholerae* strain as predator and *E. coli* prey. (C) Low c-di-GMP levels lead to interbacterial killing. *E. coli* and the indicated *V. cholerae* strains (all carrying a gene encoding a functional translation fusion between *tfoY* and mCherry at the indigenous *tfoY* locus) were co-incubated on arabinose-lacking (-ara) or arabinose-containing (+ara) LB agar plates. (D) Expression of the phosphodiesterase *cdpA* results in TfoY-dependent interbacterial killing. *E. coli* and the indicated *V. cholerae* strains (WT or *tfoY*-minus mutant ΔtfoY) expressing a diguanylate cyclase (Tn-*vdcA*) or a phosphodiesterase (Tn-*cdpA*) were co-incubated on arabinose-containing (+ara) LB agar plates. Details as in Figure 1. (E) Detection of TfoY-mCherry produced by c-di-GMP phosphodiesterase-expressing cells. *VdcA* or *cdpA* expression was induced by arabinose where indicated. The WT strain, which lacks the *tfoY-mCherry* fusion construct, served as negative control. Western blot analysis was performed as described for Figure 3C except that mCherry-specific antibodies were utilized. A nonspecific cross-reaction (marked with an asterisk) serves as a loading control. (F) Hemolytic activity of the indicated strains was tested on blood agar plates. The WT and an *hlyA*-mutant served as the positive and negative control, respectively. (G) Activity of the lecithinase (thermolabile hemolysin) was assessed on egg yolk plates. The WT and a *lec*-minus strain served as controls.

Table S1: RNA-seq expression data for the T6SS gene clusters in WT compared to TfoX#- and TfoY-induced cells.

cluster names	gene names	ID (locus tag; Heidelberg *et al.*, 2000)	A1552 (WT) +ara_Exp1	A1552 (WT) +ara_Exp2	A1552 (WT) +ara_Exp3	AVG A1552 (WT) +ara	A1552-TntfoX+ara_Exp1 (Borgeaud *et al.*, 2015)	A1552-TntfoX+ara_Exp2 (Borgeaud *et al.*, 2015)	A1552-TntfoX+ara_Exp3 (Borgeaud *et al.*, 2015)	AVG A1552-TntfoX+ara (Borgeaud *et al.*, 2015)	TfoX-induced (TfoX+ / WT)	A1552-TntfoY +ara_Exp1	A1552-TntfoY +ara_Exp2	A1552-TntfoY +ara_Exp3	AVG A1552-TntfoY +ara	TfoY-induced (TfoY+ / WT)
	tfoX	**VC1153**	8128.63	9285.04	10233.82	9215.83	29170.99	25425.36	25921.82	26839.39	2.91	5798.01	6251.69	6184.87	6078.19	0.66
	tfoY	**VC1722**	7491.01	6985.98	7931.44	7469.48	996.62	1176.54	1354.46	1175.87	0.16	31298.07	33495.54	35071.18	33288.26	4.46
Auxiliary cluster 1	*hcp1*	VC1415	120.58	137.72	86.41	114.90	4772.38	6025.73	3508.95	4769.02	41.51	3356.54	3977.41	3539.18	3624.38	31.54
	vgrG1	VC1416	108.31	106.99	103.51	106.27	1263.24	1134.86	1042.16	1146.75	10.79	620.53	761.19	710.67	697.46	6.56
	tap1	VC1417	124.66	97.88	118.81	113.78	585.63	471.75	444.47	500.62	4.40	470.19	491.62	527.85	496.56	4.36
	tseL	VC1418	488.43	503.06	586.85	526.11	2287.81	2180.67	1810.62	2093.03	3.98	3503.05	3966.05	4746.79	4071.96	7.74
	tsiV1	VC1419	92.99	124.06	126.01	114.35	654.32	921.72	658.51	744.85	6.51	794.65	958.46	1323.49	1025.53	8.97
Auxiliary cluster 2	*hcp2*	VCA0017	135.90	159.34	87.31	127.52	4629.17	5281.16	3434.10	4448.14	34.88	3432.48	3975.34	3540.46	3649.43	28.62
	vgrG2	VCA0018	18.39	19.35	22.50	20.08	482.01	404.49	449.15	445.22	22.17	203.26	231.35	140.33	191.65	9.54
	vasW	VCA0019	34.74	35.28	27.00	32.34	271.28	338.18	290.07	299.84	9.27	648.14	678.57	687.49	671.40	20.76
	vasX	VCA0020	346.40	359.66	303.32	336.46	1744.09	1784.70	1512.36	1680.38	4.99	2604.85	2566.57	2630.24	2600.55	7.73
	tsiV2	VCA0021	271.81	319.82	263.72	285.12	684.60	862.04	695.94	747.53	2.62	1007.12	1128.88	1203.76	1113.25	3.90
	PAAR1	VCA0105	83.79	78.53	129.61	97.31	357.43	231.14	266.68	285.08	2.93	258.49	296.42	239.46	264.79	2.72
		VCA0106	91.96	100.16	144.01	112.04	618.23	434.81	402.36	485.13	4.33	316.79	369.75	418.42	368.32	3.29
Large/ major cluster	*vipA*	VCA0107	50.07	59.18	47.70	52.32	1341.25	1093.18	1058.53	1164.32	22.25	418.03	515.38	525.28	486.23	9.29
	vipB	VCA0108	180.86	187.80	180.91	183.19	3384.56	2519.80	2610.66	2838.34	15.49	1337.71	1502.76	1396.87	1412.45	7.71
		VCA0109	56.20	76.26	46.80	59.75	1068.81	747.41	855.02	890.41	14.90	493.20	572.18	431.29	498.89	8.35
	vasA	VCA0110	120.58	118.37	123.31	120.75	1586.91	1307.26	1319.37	1404.51	11.63	730.98	686.83	757.02	724.94	6.00
	vasB	VCA0111	33.72	35.28	29.70	32.90	626.38	591.11	487.74	568.41	17.28	257.72	322.24	294.82	291.60	8.86
	fha	VCA0112	84.81	87.64	81.91	84.79	1009.43	842.14	884.26	911.94	10.76	520.82	549.46	608.96	559.75	6.60
	vasD	VCA0113	75.62	66.01	79.21	73.61	664.80	632.79	542.72	613.44	8.33	418.80	466.84	442.88	442.84	6.02
	vasE	VCA0114	87.88	96.74	87.31	90.64	839.44	1128.23	935.72	967.80	10.68	579.88	679.60	576.77	612.08	6.75
	vasF	VCA0115	43.94	51.22	39.60	44.92	465.71	521.01	479.56	488.76	10.88	312.18	327.41	293.54	311.04	6.92
	clpV	VCA0116	289.18	332.34	274.52	298.68	1832.57	2443.07	2014.14	2096.59	7.02	1326.97	1808.48	1462.53	1532.66	5.13
	vasH	VCA0117	197.21	253.81	244.82	231.95	1305.16	1833.96	1505.34	1548.15	6.67	1062.34	1369.53	1180.58	1204.15	5.19
	vasI	VCA0118	39.85	46.66	29.70	38.74	310.86	440.49	333.35	361.57	9.33	233.95	235.48	215.00	228.14	5.89
	vasJ	VCA0119	139.99	166.17	117.91	141.36	773.08	1092.23	895.95	920.42	6.51	741.72	941.94	749.29	810.98	5.74
	vasK	VCA0120	525.22	680.61	460.84	555.56	2522.99	3768.33	2609.49	2966.94	5.34	2324.88	3200.72	2822.07	2782.56	5.01
	vasL	VCA0121	330.05	374.45	288.02	330.84	936.08	1589.56	1180.18	1235.27	3.73	1122.17	1591.58	1350.53	1354.76	4.09
	vasM	VCA0122	38.83	34.14	33.30	35.43	64.04	92.83	101.76	86.21	2.43	92.81	124.97	124.88	114.22	3.22
	vgrG3	VCA0123	790.89	931.01	806.46	842.79	1636.97	2488.54	1864.42	1996.65	2.37	1963.61	2550.04	2471.89	2328.51	2.76
	tsiV3	VCA0124	345.38	586.15	477.94	469.82	826.64	1108.33	800.04	911.67	1.94	715.64	1093.76	1084.03	964.48	2.05
Auxiliary cluster 3	*PAAR2*	VCA0284	157.36	166.17	284.42	202.65	235.18	222.61	216.39	224.73	1.11	293.77	361.49	379.80	345.02	1.70
	tseH	VCA0285	182.91	146.82	275.42	201.72	308.53	294.61	271.36	291.50	1.45	423.40	424.49	561.32	469.74	2.33
	tsiH	VCA0286	161.45	129.75	221.42	170.87	239.84	306.92	232.76	259.84	1.52	505.48	527.77	690.07	574.44	3.36

TfoX-induced data are from a previous study (Borgeaud et al., 2015).

Table S2: Bacterial cytotoxicity against the amoeba *Dictyostelium discoideum*

	Numbers of plaques[#] (±SD)	Statistics compared to strain V52Δrhh	Relative population size compared to *Klebsiella* negative control
V52Δrhh	17 (± 11)		10.2%
V52ΔrhhΔtfoY	159 (± 25)	** (p = 0.00619)	95.2%
Klebsiella sp.	167 (± 18)	** (p = 0.00129)	100%

Average number of plaques from three independent experiments with three replicates each ± standard deviation (SD).

Table S3: ***V. cholerae*** **strains and plasmids used in this study**

Strains or Plasmid	Genotype*/description	Internal strain No	Reference
A1552 (WT)	Wild-type, O1 El Tor Inaba; Rif^R	MB_1	(Yildiz and Schoolnik, 1998)
A1552-Tn*tfoX-strep*	A1552 containing mini-Tn7-*araC*-P_{BAD}-*tfoX-strep*; Rif^R, $Gent^R$	MB_3420	This study
A1552-Tn*tfoY*	A1552 containing mini-Tn7-*araC*-P_{BAD}-*tfoY*; Rif^R, $Gent^R$	MB_2979	This study
A1552-Tn*tfoY-strep*	A1552 containing mini-Tn7-*araC*-P_{BAD}-*tfoY-strep*; Rif^R, $Gent^R$	MB_2978	This study
A1552-Tn*tfoY-mCherry-strep*	A1552 containing mini-Tn7- *araC*-P_{BAD}-*tfoY-mCherry-strep*; Rif^R, $Gent^R$	MB_4322	This study
A1552-Tn-*vdcA*	A1552 containing mini-Tn7-*araC*-P_{BAD}-*vdcA*; Rif^R, $Gent^R$	MB_2947	This study
A1552-Tn-*cdpA*	A1552 containing mini-Tn7-*araC*-P_{BAD}-*cdpA*; Rif^R, $Gent^R$	MB_2948	This study
A1552ΔhapR	A1552ΔVC0583; Rif^R	MB_3	(Meibom et al., 2005)
A1552ΔhapR-Tn*tfoY-strep*	A1552ΔhapR containing mini-Tn7- *araC*-P_{BAD}-*tfoY-strep*; Rif^R, $Gent^R$	MB_4267	This study
A1552ΔhlyA	A1552 deleted for *VCA0219* (TransFLP); Rif^R	MB_3935	This study
A1552Δlec	A1552 deleted for *VCA0218* (TransFLP); Rif^R	MB_4189	This study
A1552ΔqstR	A1552ΔVC0396; Rif^R	MB_600	(Lo Scrudato and Blokesch, 2013)
A1552ΔqstR-Tn*tfoY-strep*	A1552ΔqstR containing mini-Tn7- *araC*-P_{BAD}-*tfoY-strep*; Rif^R, $Gent^R$	MB_3772	This study
A1552ΔrpoN	A1552 deleted for *VC2529* (TransFLP); Rif^R	MB_4034	This study
A1552ΔrpoN-Tn*tfoX-strep*	A1552ΔrpoN (TransFLP) containing mini-Tn7- *araC*-P_{BAD}-*tfoX-strep*; Rif^R, $Gent^R$	MB_4088	This study
A1552ΔrpoN-Tn*tfoY-strep*	A1552ΔrpoN (TransFLP) containing mini-Tn7- *araC*-P_{BAD}-*tfoY-strep*; Rif^R, $Gent^R$	MB_4089	This study
A1552ΔtfoX	A1552ΔtfoX (TransFLP); Rif^R	MB_1447	(Borgeaud et al., 2015)
A1552ΔtfoX-Tn*tfoY-strep*	A1552ΔtfoX (TransFLP) containing mini-Tn7- *araC*-P_{BAD}-*tfoY-strep*; Rif^R, $Gent^R$	MB_3418	This study
A1552ΔtfoY	A1552ΔVC1722 (deleted using suicide plasmid pGP704-28-SacB-Δ*tfoY*); Rif^R	MB_828	This study
A1552ΔtfoY-Tn*tfoX-strep*	A1552ΔtfoY containing mini-Tn7- *araC*-P_{BAD}-*tfoX-strep*; Rif^R, $Gent^R$	MB_3419	This study
A1552ΔtfoY-Tn-*vdcA*	A1552ΔtfoY containing mini-Tn7- *araC*-P_{BAD}-*vdcA*; Rif^R, $Gent^R$	MB_3945	This study
A1552ΔtfoY-Tn-*cdpA*	A1552ΔtfoY containing mini-Tn7- *araC*-P_{BAD}-*cdpA*; Rif^R, $Gent^R$	MB_3946	This study
A1552ΔtseHΔtsiH	A1552 deleted for *VCA0285* and *VCA0286* (TransFLP); Rif^R	MB_4131	This study
A1552ΔtseHΔtsiH-Tn*tfoX-strep*	A1552ΔtseHΔtsiH (TransFLP) containing mini-Tn7- *araC*-P_{BAD}-*tfoX-strep*; Rif^R, $Gent^R$	MB_4185	This study
A1552ΔtseHΔtsiH-Tn*tfoY-strep*	A1552ΔtseHΔtsiH (TransFLP) containing mini-Tn7- *araC*-P_{BAD}-*tfoY-strep*; Rif^R, $Gent^R$	MB_4186	This study
A1552ΔvasH	A1552 deleted for *VCA0117* (TransFLP); Rif^R	MB_3928	This study
A1552ΔvasH-Tn*tfoX-strep*	A1552ΔvasH (TransFLP) containing mini-Tn7- *araC*-P_{BAD}-*tfoX-strep* -strep; Rif^R, $Gent^R$	MB_3936	This study
A1552ΔvasH-Tn*tfoY-strep*	A1552ΔvasH (TransFLP) containing mini-Tn7- *araC*-P_{BAD}-*tfoY-strep*; Rif^R, $Gent^R$	MB_3937	This study
A1552ΔvasK	A1552ΔVCA0120; Rif^R	MB_585	(Borgeaud et al., 2015)

Strains or Plasmid	Genotype*/description	Internal strain No	Reference
A1552ΔvasK-Tn*tfoY-strep*	A1552ΔvasK containing mini-Tn7- *araC*-P_{BAD}-*tfoY-strep*; Rif^R, $Gent^R$	MB_3578	This study
A1552ΔvipA	A1552ΔVCA0107 (TransFLP); Rif^R	MB_3042	(Borgeaud et al., 2015)
A1552ΔvipA-Tn*tfoY-strep*	A1552ΔvipA containing mini-Tn7- *araC*-P_{BAD}-*tfoY-strep*; Rif^R, $Gent^R$	MB_3580	This study
A1552ΔvipB	A1552ΔVCA0108; Rif^R	MB_598	(Borgeaud et al., 2015)
A1552ΔvipB-Tn*tfoY-strep*	A1552ΔvipB containing mini-Tn7- *araC*-P_{BAD}-*tfoY-strep*; Rif^R, $Gent^R$	MB_3579	This study
A1552-tfoY-mCherry	A1552 carrying *tfoY-mCherry* translational fusion (TransFLP); Rif^R	MB_4262	This study
A1552-tfoY-mCherry-Tn-*vdcA*	A1552-tfoY-mCherry containing mini-Tn7- *araC*-P_{BAD}-*vdcA*; Rif^R, $Gent^R$	MB_4324	This study
A1552-tfoY-mCherry-Tn-*cdpA*	A1552-tfoY-mCherry containing mini-Tn7- *araC*-P_{BAD}-*cdpA*; Rif^R, $Gent^R$	MB_4325	This study
A1552-vipA-sfGFPv2	A1552 carrying *vipA-sfgfp* translational fusion (TransFLP), as previously described (Borgeaud et al., 2015); this fusion version (v2) has 3 bp of the linker region removed; Rif^R	MB_3909	This study
A1552-vipA-sfGFPv2-Tn*tfoX-strep*	A1552-vipA-sfGFPv2 containing mini-Tn7- *araC*-P_{BAD}-*tfoX-strep*; Rif^R, $Gent^R$	MB_3961	This study
A1552-vipA-sfGFPv2-Tn*tfoY-strep*	A1552-vipA-sfGFPv2 containing mini-Tn7 *araC*-P_{BAD}-*tfoY-strep* Rif^R, $Gent^R$	MB_3962	This study
A1552ΔhapR-vipA-sfGFPv2	A1552-vipA-sfGFPv2 (TransFLP) ΔVC0583 (deleted using suicide plasmid pGP704-28-SacB-Δ*hapR*); Rif^R	MB_4217	This study
A1552ΔhapR-vipA-sfGFPv2-Tn*tfoX-strep*	A1552ΔhapR-vipA-sfGFPv2 containing mini-Tn7- *araC*-P_{BAD}-*tfoX-strep*; Rif^R, $Gent^R$	MB_4261	This study
A1552ΔhapR-vipA-sfGFPv2-Tn*tfoY-strep*	A1552ΔhapR-vipA-sfGFPv2 containing mini-Tn7- *araC*-P_{BAD}-*tfoY-strep*; Rif^R, $Gent^R$	MB_4260	This study
SA5YΔvipA-*dsRED*	SA5YΔvipA-*dsRED;* Kan^R, $Gent^R$	MB_3052	(Borgeaud et al., 2015)
V52Δrhh	V52Δrhh; Str^R	MB_3778	(Basler et al., 2012)
V52Δrhh-Tn*tfoY-strep*	V52Δrhh containing mini-Tn7- *araC*-P_{BAD}-*tfoY-strep*; Str^R, $Gent^R$	MB_4209	This study
V52ΔrhhΔtfoY	V52ΔrhhΔVC1722 (deleted using suicide plasmid pGP704-28-SacB-Δ*tfoY*); ; Str^R	MB_4179	This study
V52ΔrhhΔtfoY-Tn*tfoY-strep*	V52ΔrhhΔtfoY containing mini-Tn7- *araC*-P_{BAD}-*tfoY-strep*; Str^R, $Gent^R$	MB_4211	This study
V52ΔrhhΔtfoX	V52ΔrhhΔVC1153 (deleted using suicide plasmid pGP704-28-SacB-Δ*tfoX*); Str^R	MB_4383	This study
***E. coli* & others**			
SM10λpir	thi-1 thr leu tonA lacY supE recA::RP4-2-Tc::Mu, Kmr (λpir); Kan^R	MB_647	(Simon et al., 1983)
TOP10	F- mcrA Δ(mrr-hsdRMS-mcrBC) φ80lacZΔM15 ΔlacX74 nupG recA1 araΔ139 Δ(ara-leu)7697 galE15 galK16 rpsL(Str^R) endA1λ^-	MB_741	Invitrogen
TOP10-TnKan	TOP10 containing mini-Tn7-*aph* (Kan^R); Str^R, Kan^R, $Gent^R$	MB_4119	This study
DH5α	F^- endA1 glnV44 thi-1 recA1 relA1 gyrA96 deoR nupG φ80lacZΔM15 Δ(lacZYA-argF) U169 hsdR17 (r_K^- m_K^+) phoA, λ-	MB_736	(Yanisch-Perron et al., 1985)
S17-1λpir	Tpr Smr recA thi pro hsdR2M1 RP4:2-Tc:Mu:Kmr Tn7 (λpir); Str^R	MB_648	(Simon et al., 1983)

Strains or Plasmid	Genotype*/description	Internal strain No	Reference
Klebsiella sp.	*Klebsiella* species; *gfp*$^+$	MB_4331	(Benghezal et al., 2006) (P. Cosson, Geneva)
**Plasmids**			
pBAD/myc-HisA	pBR322-derived expression vector; *araBAD* promoter (P_{BAD}); Amp^R	MB_24	Invitrogen
pBAD-*tfoX-strep*	*tfoX* in pBAD/Myc-HisA with C-terminal *Strep*-tagII, arabinose inducible; Amp^R	MB_3616	This study
pBAD-*tfoY*	*tfoY* in pBAD/Myc-HisA without tag, arabinose inducible; Amp^R	MB_768	This study
pBAD-*tfoY-strep*	*tfoY* in pBAD/Myc-HisA with C-terminal *Strep*-tagII, arabinose inducible; Amp^R	MB_2945	This study
pBAD-*tfoY-strep*-CSmaI	pBAD-*tfoY-strep* including SmaI site between tfoY gene and *Strep*-tagII; Amp^R	MB_4385	This study
pBAD-*tfoY-mCherry*-strep	*tfoY-mCherry* (translational fusion) in pBAD/Myc-HisA with C-terminal *Strep*-tagII, arabinose inducible; Amp^R	MB_4386	This study
pBAD-*vdcA*	*vdcA* in pBAD/Myc-HisA without tag, arabinose inducible; Amp^R	MB_2066	This study
pBAD-*cdpA*	*cdpA* in pBAD/Myc-HisA without tag, arabinose inducible; Amp^R	MB_2065	This study
pGP704-mTn7-minus SacI	pGP704 with mini-*Tn*7; Amp^R, $Gent^R$	MB_645	(Müller et al., 2007)
pGP704-mTn*tfoX-strep*	pGP704 with mini-Tn7 carrying *araC* and P_{BAD}-driven *tfoX-strep*; Amp^R, $Gent^R$	MB_3664	This study
pGP704-mTn*tfoY*	pGP704 with mini-Tn7 carrying *araC* and P_{BAD}-driven *tfoY*; Amp^R, $Gent^R$	MB_2953	This study
pGP704-mTn*tfoY-strep*	pGP704 with mini-Tn7 carrying *araC* and P_{BAD}-driven *tfoY-strep*; Amp^R, $Gent^R$	MB_2941	This study
pGP704-mTn*tfoY-mCherry-strep*	pGP704 with mini-Tn7 carrying *araC* and P_{BAD}-driven *tfoY-mCherry-strep*; Amp^R, $Gent^R$	MB_4387	This study
pGP704-mTn-*vdcA*	pGP704 with mini-Tn7 carrying *araC* and P_{BAD}-driven *vdcA*; Amp^R, $Gent^R$	MB_2943	This study
pGP704-mTn-*cdpA*	pGP704 with mini-Tn7 carrying *araC* and P_{BAD}-driven *cdpA*; Amp^R, $Gent^R$	MB_2944	This study
pGP704-TnKan	pGP704 with mini-Tn7 carrying *aph* (Kan^R) gene; Amp^R, $Gent^R$, Kan^R	MB_4117	This study
pUX-BF-13	*ori*R6K, helper plasmid with Tn7 transposition function; Amp^R	MB_457	(Bao et al., 1991)
pGP704-Sac28	Suicide vector, *ori*R6K, *sacB*; Amp^R	MB_649	(Meibom et al., 2004)
pGP704-28-SacB-Δ*tfoY*	pGP704-Sac28 with gene fragment resulting in a 180 bp deletion within *VC1722*; Amp^R	MB_1133	This study
p28-hapR (pGP704-28-SacB-Δ*hapR*)	pGP704-Sac28 with gene fragment resulting in a deletion from amino acid 10 downwards of *hapR;* Amp^R	MB_1106	(Meibom et al., 2005)
p28-tfoX (pGP704-28-SacB-Δ*tfoX*)	pGP704-Sac28 with a gene fragment resulting in a deletion of amino acids 6-196 of TfoX; Amp^R	MB_1013	This study
pBR-FRT-Kan-FRT2	pBR322 derivative containing improved FRT-*aph*-FRT cassette, used as template for TransFLP; Amp^R, Kan^R	MB_3782	This study
pBR-FRT-Cat-FRT2	pBR322 derivative containing improved FRT-*cat*-FRT cassette, used as template for TransFLP; Amp^R, Cm^R	MB_3783	This study

*VC numbers according to (Heidelberg et al., 2000).

EXTENDED EXPERIMENTAL PROCEDURES

Bacterial strains, plasmids, and growth conditions

V. cholerae strains and plasmids are listed in Table S3. *Escherichia coli* strains DH5α (Yanisch-Perron et al., 1985), TOP10 (Invitrogen), and S17-1λpir (Simon et al., 1983) were used for cloning purposes and served as donors in bacterial mating experiments, respectively. A V52Δrhh (Δ*rtxA* Δ*hlyA* Δ*hapA*) strain served as the parental strain for T6SS activity and amoebal killing (see below), in accordance with previous protocols (Bachmann et al., 2015; Miyata et al., 2011).

V. cholerae and *E. coli* strains were grown aerobically in LB medium or on LB agar plates at 30°C or 37°C unless otherwise stated. LB motility soft agar plates contained reduced agar concentrations (0.3%) compared to standard LB agar plates (1.5%). When required, arabinose (for the expression of *tfoX*, *tfoY, tfoY-mCherry, vdcA*, and *cpdA* under the control of the P_{BAD} promoter) or antibiotics were added to the growth medium at the following concentrations: L-arabinose 0.02% or 0.2%, ampicillin 50 μg/ml or 100 μg/ml, kanamycin 75 μg/ml, streptomycin 100 μg/ml, chloramphenicol 2.5 μg/ml, and gentamicin 50 μg/ml. For *E. coli* counter-selection after tri-parental mating with *V. cholerae,* Thiosulfate Citrate Bile Salts Sucrose (TCBS) agar plates were used and prepared following the manufactures' instructions (Sigma-Aldrich/Fluka, Buchs, Switzerland). Half-concentration defined artificial seawater (0.5x DASW; (Meibom et al., 2005)) was used for chitin-induced natural transformation experiments.

Genetic engineering of strains and plasmids

All DNA manipulations were performed according to standard molecular biology protocols. Enzymes were purchased from the following companies and used as recommended by the manufacturer: Pwo polymerase (Roche), Taq polymerase (Promega), and restriction enzymes (New England Biolabs). Genetically engineered bacterial strains and plasmids were screened by colony PCR followed by Sanger sequencing (Microsynth, Switzerland).

To genetically modify *V. cholerae* strains, a gene disruption method based on the counter-selectable suicide plasmid pGP704-Sac28 (Meibom et al., 2004) or a transformation-based genetic engineering method (TransFLP; (Blokesch, 2012; Borgeaud and Blokesch, 2013; De Souza Silva and Blokesch, 2010)) was utilized. The latter technique was also used to replace the *tfoY* gene of *V. cholerae* A1552 with a translational *tfoY-mCherry* fusion allele at the gene's native locus. The plasmids pBR-FRT-Kan-FRT2 and pBR-FRT-Cat-FRT2 served as templates for the addition of the flippable antibiotics cassette.

Plasmid pBAD-*tfoX-strep* was constructed by amplification of the *tfoX* gene with *Strep-tagII*-encoding primers using the genomic DNA (gDNA) of *V. cholerae* strain A1552 as the template. The restriction enzyme-digested PCR product was cloned into the appropriately digested plasmid pBAD/MycHisA (Table S3). The *tfoY* gene was amplified using gDNA from *V. cholerae* strain A1552 and cloned after restriction enzyme digestion into the plasmid pBAD/MycHisA, resulting in plasmid pBAD-*tfoY* (Table S3). The same strategy was employed to obtain plasmids pBAD-*vdcA* and pBAD-*cdpA* (Table S3). The *Strep-tagII*-encoding sequence was added to *tfoY* by inverse PCR using overlapping oligonucleotides. The template was subsequently digested by *Dpn*I, and the non-digested PCR fragment was column-purified and used directly to transform chemically competent *E. coli* cells.

The plasmid pBAD-*tfoY-mCherry-strep* was constructed in two steps. First, a *Sma*I restriction site was introduced in-between the *tfoY* open reading frame and the *Strep-tagII* by inverse PCR on plasmid pBAD-*tfoY-strep,* as described above, resulting in plasmid pBAD-*tfoY-strep*-CSmaI. Subsequently, the mCherry-encoding gene was amplified using phosphorylated primers and cloned into the *Sma*I-digested pBAD-*tfoY-strep*-CSmaI plasmid.

The fragments containing *araC*, the arabinose-inducible promoter P_{BAD}, and the *tfoX-strep*, *tfoY-strep, tfoY-mCherry-strep*, *vdcA* or *cdpA* gene were amplified from the corresponding pBAD plasmids (Table S3) and cloned into the mini-Tn7-containing delivery plasmid (Table S3). For the insertion of this mini-Tn7 transposon into the *V. cholerae* chromosome, a triparental mating strategy was employed (Bao et al., 1991). The donor plasmids are indicated in Table S3.

Natural transformation assays

The natural transformability of *V. cholerae* strains grown on chitin surfaces was determined as previously described (De Souza Silva and Blokesch, 2010; Marvig and Blokesch, 2010). To test for chitin-independent transformation, the strains carried an arabinose-inducible copy of *tfoX-strep* (e.g., including the *Strep*-tagII-encoding sequence) or *tfoY-strep* on the chromosome. The assay was performed as previously described (Lo Scrudato and Blokesch, 2012). The gDNA of A1552-lacZ-Kan (Marvig and Blokesch, 2010) served as the transforming material in all transformation assays. Transformation frequencies were calculated as the number of transformants divided by the total number of colony-forming units (CFU). At least three independent biological replicates were performed for

each experiment, and averages of all experiments are indicated in the figure (±SD). Statistical analyses of transformation frequencies were performed on log-transformed data using a two-tailed Student's *t*-test. For values below the detection limit, the detection limit value was utilized for statistical calculations.

Interbacterial killing assay

The interbacterial killing assay was performed following a previously established protocol (Borgeaud et al., 2015). Briefly, the predatory *V. cholerae* strain and the *E. coli* prey were mixed at a 10:1 ratio and spotted onto membrane filters on pre-warmed LB agar plates (± 0.2% ara). After incubation at 37°C for 4 h, the bacteria were resuspended from the filters, and serial dilutions were spotted onto plain LB (growth control) and antibiotic-containing LB agar plates (to select for the respective *E. coli* prey strains) to enumerate colony-forming units (CFU/ml). At least three biological experiments were performed, and averages of these independent replicates are given throughout the manuscript. Statistically significant differences were determined by the two-tailed Student's *t*-test on log-transformed data. In case the *E. coli* CFUs were below the detection limit of 200 CFU/ml, the value was set to the detection limit to allow statistical analysis.

Motility assay

To assess the motility of *V. cholerae,* 2 μl of the relevant overnight culture was carefully spotted onto freshly prepared LB motility agar plates and incubated at room temperature for 24 h. The next day, the swarming diameter was scored. The motility induction was calculated by dividing the swarming diameter under induced versus uninduced conditions. All experiments were repeated several times independently ($n \geq 3$), and averages of all experiments (± standard deviation) are provided. A two-tailed Student's *t*-test was performed for statistical analyses.

Gene expression analysis by qRT-PCR

Quantitative reverse transcription PCR (qRT-PCR)-based transcript scoring in *V. cholerae* was performed following a previously published protocol (Lo Scrudato and Blokesch, 2012). Averages of at least three biologically independent experiments (± standard deviation) are provided.

SDS-PAGE and Western Blotting

For the preparation of cell lysates, bacteria were cultivated in LB medium without or with arabinose, as indicated. After harvesting, the bacterial cells were resuspended in an appropriate volume of Laemmli buffer to adjust for the total number of bacteria (based on measurement of the optical density at 600 nm; OD_{600}) and boiled for 15 minutes. Proteins were separated by sodium dodecyl sulfate (SDS)-polyacrylamide gel electrophoresis and subjected to western blotting as previously described (Lo Scrudato and Blokesch, 2012). Primary antibodies against Hcp (see below), *E. coli* Sigma 70 (BioLegend, USA distributed via Brunschwig, Switzerland), or mCherry (BioVision, USA distributed via LubioScience, Switzerland) were diluted at 1:5'000. Goat anti-rabbit horseradish peroxidase (HRP) (diluted 1:20'000; Sigma-Aldrich, Switzerland) and goat anti-mouse HRP (diluted 1:5'000, Sigma-Aldrich, Switzerland) were used as secondary antibodies. Lumi-Light^{PLUS} western blotting substrate (Roche, Switzerland) was used as the HRP substrate and the signals were detected on chemiluminescence-detecting films (Amersham Hyperfilm ECL, GE Healthcare).

Generation of antibodies against Hcp

Anti-Hcp antibodies were raised in rabbits against synthetic peptides (Eurogentec, Belgium). To exclude potential cross-reactions with proteins migrating towards the same position as the target protein, the antibody was tested against the *hcp1 hcp2* double knockout strain using western blotting (data not shown).

Epifluorescence microscopy and image analysis

Wide-field microscopy images were acquired using an epifluorescence microscope (Zeiss Axio Imager M2; details are provided elsewhere (Lo Scrudato and Blokesch, 2012)). The bacterial cultures were grown in LB medium (±ara) and then mounted onto agarose pads. Image analysis and processing were performed using the Zeiss AxioVision software, ImageJ, and Adobe Illustrator. For comparisons between different culture conditions (e.g., WT versus *hapR*-minus strains grown in the absence or presence of inducible *tfoX* or *tfoY*), the images were acquired using the exact same exposure time. However, the brightness was decreased for the TfoX-induced vipA-sfGFP signal for a better representation. For intraspecies killing, the two strains (predator and non-immune prey) were mixed at a ratio of 1:1 before being applied to an agarose pad. The mixed communities were incubated for 2 h at 24°C before imaging.

***Dictyostelium discoideum* plaque assay**

To determine the cytotoxicity of *V. cholerae* strain V52 (V52Δrhh) toward *D. discoideum*, an amoebal plaque assay was performed following a previously described protocol (Pukatzki et al., 2006) with slight modifications. Briefly, bacteria were cultured overnight in LB medium at 30°C and harvested by centrifugation. The cell pellet was resuspended in SorC buffer and diluted to an OD_{600} of 5.5. *D. discoideum* cells were detached, collected by centrifugation, resuspended in SorC buffer, and added to the bacterial suspension to a concentration of 5 x 10^2 amoebal cells per ml. Subsequently, 200 µl of the homogenate was plated on agar plates made of 5-fold diluted SM medium (SM medium according to (Sussman, 1987)). Plates were incubated for 3 to 5 days at 24°C, followed by the enumeration of the number of *D. discoideum* plaques. Three independent experiments were performed, each containing three technical replicates, and a two-tailed Student's *t*-test was performed to ascertain statistical significance.

Hemolysin activity on blood agar plates

The hemolytic activity of *V. cholerae* was assayed using trypticase soy agar containing 5% sheep blood (BD, Allschwil, Switzerland) supplemented with 1 mM EDTA. The respective overnight cultures were spotted onto the plates and incubated at 30°C for 24 h to 30 h. The WT and a *hlyA*-minus mutant served as the positive and negative control, respectively.

Lecithinase / thermolabile hemolysin activity on egg yolk plates

To assess the activity of the lecithinase (also known as thermolabile hemolysin/phospholipase), *V. cholerae* bacteria grown overnight were spotted onto egg yolk-containing agar plates and incubated at 30°C for 72 h. Plates were freshly prepared as follows: a 30% egg yolk (Sigma-Aldrich/Fluka, Buchs, Switzerland) suspension in sterile water was prepared and subsequently diluted 1:20 in autoclaved, warm LB agar before plates were poured. The WT and a *lec*-minus mutant strain served as the positive and negative control, respectively.

SUPPLEMENTAL REFERENCES

Bachmann, V., Kostiuk, B., Unterweger, D., Diaz-Satizabal, L., Ogg, S., and Pukatzki, S. (2015). Bile Salts Modulate the Mucin-Activated Type VI Secretion System of Pandemic *Vibrio cholerae*. PLoS Negl. Trop. Dis. *9*, e0004031.

Bao, Y., Lies, D.P., Fu, H., and Roberts, G.P. (1991). An improved Tn*7*-based system for the single-copy insertion of cloned genes into chromosomes of Gram-negative bacteria. Gene *109*, 167-168.

Basler, M., Pilhofer, M., Henderson, G.P., Jensen, G.J., and Mekalanos, J.J. (2012). Type VI secretion requires a dynamic contractile phage tail-like structure. Nature *483*, 182-186.

Benghezal, M., Fauvarque, M.O., Tournebize, R., Froquet, R., Marchetti, A., Bergeret, E., Lardy, B., Klein, G., Sansonetti, P., Charette, S.J., *et al.* (2006). Specific host genes required for the killing of *Klebsiella* bacteria by phagocytes. Cell. Microbiol *8*, 139-148.

Blokesch, M. (2012). TransFLP – a method to genetically modify *V. cholerae* based on natural transformation and FLP-recombination. J. Vis. Exp. *68*, e3761.

Borgeaud, S., and Blokesch, M. (2013). Overexpression of the *tcp* gene cluster using the T7 RNA polymerase/promoter system and natural transformation-mediated genetic engineering of *Vibrio cholerae*. PLoS One *8*, e53952.

Borgeaud, S., Metzger, L.C., Scrignari, T., and Blokesch, M. (2015). The type VI secretion system of *Vibrio cholerae* fosters horizontal gene transfer. Science *347*, 63-67.

De Souza Silva, O., and Blokesch, M. (2010). Genetic manipulation of *Vibrio cholerae* by combining natural transformation with FLP recombination. Plasmid *64*, 186-195.

Heidelberg, J.F., Eisen, J.A., Nelson, W.C., Clayton, R.A., Gwinn, M.L., Dodson, R.J., Haft, D.H., Hickey, E.K., Peterson, J.D., Umayam, L., *et al.* (2000). DNA sequence of both chromosomes of the cholera pathogen *Vibrio cholerae*. Nature *406*, 477-483.

Lo Scrudato, M., and Blokesch, M. (2012). The regulatory network of natural competence and transformation of *Vibrio cholerae*. PLoS Genet. *8*, e1002778.

Lo Scrudato, M., and Blokesch, M. (2013). A transcriptional regulator linking quorum sensing and chitin induction to render *Vibrio cholerae* naturally transformable. Nucleic Acids Res. *41*, 3644-3658.

Marvig, R.L., and Blokesch, M. (2010). Natural transformation of *Vibrio cholerae* as a tool-optimizing the procedure. BMC Microbiol. *10*, 155.

Meibom, K.L., Blokesch, M., Dolganov, N.A., Wu, C.-Y., and Schoolnik, G.K. (2005). Chitin induces natural competence in *Vibrio cholerae*. Science *310*, 1824-1827.

Meibom, K.L., Li, X.B., Nielsen, A.T., Wu, C.Y., Roseman, S., and Schoolnik, G.K. (2004). The *Vibrio cholerae* chitin utilization program. Proc. Natl. Acad. Sci. USA *101*, 2524-2529.

Miyata, S.T., Kitaoka, M., Brooks, T.M., McAuley, S.B., and Pukatzki, S. (2011). *Vibrio cholerae* requires the type VI secretion system virulence factor VasX to kill Dictyostelium discoideum. Infect. Immun. *79*, 2941-2949.

Müller, J., Miller, M.C., Nielsen, A.T., Schoolnik, G.K., and Spormann, A.M. (2007). *vpsA*- and *luxO*-independent biofilms of *Vibrio cholerae*. FEMS Microbiol. Lett. *275*, 199-206.

Pukatzki, S., Ma, A.T., Sturtevant, D., Krastins, B., Sarracino, D., Nelson, W.C., Heidelberg, J.F., and Mekalanos, J.J. (2006). Identification of a conserved bacterial protein secretion system in *Vibrio cholerae* using the *Dictyostelium* host model system. Proc. Natl. Acad. Sci. USA *103*, 1528-1533.

Simon, R., Priefer, U., and Pühler, A. (1983). A broad host range mobilization system for *in vivo* genetic engineering: transposon mutagenesis in Gram negative bacteria. Nat. Biotechnol. *1*, 784-791.

Sussman, M. (1987). Cultivation and synchronous morphogenesis of *Dictyostelium* under controlled experimental conditions. Methods Cell Biol. *28*, 9-29.

Yanisch-Perron, C., Vieira, J., and Messing, J. (1985). Improved M13 phage cloning vectors and host strains: nucleotide sequences of the M13mp18 and pUC19 vectors. Gene *33*, 103-119.

Yildiz, F.H., and Schoolnik, G.K. (1998). Role of *rpoS* in stress survival and virulence of *Vibrio cholerae*. J. Bacteriol. *180*, 773-784.
